# Double jeopardy study protocol: mixed-methods study to understand ANHPI college students at the intersection of sexual violence and anti-Asian racism after COVID-19

**DOI:** 10.1186/s12889-025-25533-8

**Published:** 2025-12-22

**Authors:** Eunhee Park, Jianchao Lai, Rhea Gandhi, Jenny Lee, Allison Massey, Madison Logan, Grace Nguyen, Sabrina Dou, Jennifer A. Wagman

**Affiliations:** 1 Department of Community Health Sciences, Jonathan and Karin Fielding School of Public Health, University of California Los Angeles, 650 Charles E Young Dr S, Los Angeles, CA 90095 United States; 2 Department of Social Welfare, Luskin School of Public Affairs, University of California Los Angeles, Los Angeles, CA United States; 3 School of Medicine, University of California Irvine, Irvine, CA United States; 4 Luskin School of Public Affairs, University of California Los Angeles, Los Angeles, CA United States; 5 Department of Communication, College of Social Sciences, University of California Los Angeles, Los Angeles, CA United States; 6 Department of Ecology and Evolutionary Biology, College of Letters and Science, University of California Los Angeles, Los Angeles, CA United States; 7 Department of Psychology, College of Letters and Science, University of California Los Angeles, Los Angeles, CA United States

**Keywords:** Campus-based violence prevention, Intimate partner violence, Mixed methods research, Prevention, Public health approach, Sexual violence, Trauma-informed research, Participatory action research

## Abstract

**Background:**

The rise in anti-Asian hate crimes and the concurrent risk of violence against women has created an urgent need to understand the mental health and help-seeking behaviors of Asian, Native Hawaiian, and Pacific Islander (ANHPI) female college students. However, data on these intersecting issues are scarce, limiting our understanding and the availability of evidence-based advocacy tools and culturally specific services for ANHPI students who have experienced sexual violence. This mixed method study assesses experiences of sexual violence and anti-Asian racism and related mental and physical health among ANHPI students across the University of California (UC) campuses with more than 40% of the students identified as ANHPIs.

**Methods:**

The Double Jeopardy Study uses a mixed-methods approach, combining survey data, qualitative interviews, and participatory-action Transmedia-Photovoice research. These methods are informed by intersectional, community-engaged frameworks and guided by trauma-informed, survivor-centered principles. The Double Jeopardy Study employs a sequential approach of three methodologies: quantitative online surveys; qualitative in-depth interviews; and Trnasmedia-Photovoice. Data are being collected from since September 2021 across all 10 UC campuses. Data collection and analysis are ongoing.

**Discussion:**

The findings from this study will be used to inform programs aimed at preventing violence, promoting the health and well-being of ANHPI students, and enhancing services to meet the needs of ANHPI victim-survivors. Additionally, the findings will inform policies that promote culturally specific services for ANHPI student victim-survivors.

**Supplementary Information:**

The online version contains supplementary material available at 10.1186/s12889-025-25533-8.

## Background

Sexual violence is a pervasive public health problem on college and university campuses, affecting approximately one in four female students and disproportionately impacting racial minority students, both in terms of prevalence and adverse outcomes [1, 2]. Sexual violence and sexual harassment (SVSH) have immense short-term impacts on survivors’ mental and physical health including, but not limited to, physical injury, low self-esteem, depression, anxiety, post-traumatic stress disorder (PTSD) [3–7], and long-term impacts such as higher transfer and college dropout rates [8, 9] and increased risk for chronic diseases [10].

Many forms of oppression, including gender discrimination, racism, and xenophobia, increase the risk of sexual violence considering that people with less power are commonly targeted as victims of SVSH [11–13]. Anti-Asian racism and hate crimes have increased substantially since the start of the Coronavirus Disease 2019 (COVID-19) pandemic, including on college campuses [14–20]. Data from four major United States (U.S.) cities show that hate crimes against Asians surged after March 2020 [21]. Another study examines the experiences of discrimination among Asian American college students during the COVID-19 pandemic. It has been reported that women and gender minority students are significantly more likely to experience both direct and indirect discrimination compared to heterosexual male students [22]. Discriminatory beliefs and stereotypes about marginalized communities are often used to justify violence, discrimination, and acts of SVSH [23–26].

Despite the high prevalence and negative consequences of SVSH, victims-survivor^1^ often refrain from reporting incidents and seeking support. Barriers to reporting violence and seeking support are often compounded among Asian, Native Hawaiian, and Pacific Islander (ANHPI) female student victim-survivors. Across Asian cultures, stigma and rape myths are rampant, leading to increased shame for victims and impeding their ability to speak about their experiences [28, 29]. Furthermore, despite the high number of ANHPI students in the U.S. higher education system, substantial gaps have been identified in the availability of culturally specific and accessible SVSH services for ANHPI student victim-survivors [29, 30]. For example, the University of California (UC) system accounts for more than 40% of ANHPI student enrollment (see Appendix 1 for detailed student demographics in UC), however, there are limited culturally specific SVSH services for ANHPI student victim-survivors [31].

Given the unique factors involved, there is a critical gap in the literature assessing SVSH and the factors affecting help-seeking behavior among ANHPI student victim-survivors in the U.S. higher education [29, 30]. Since the start of the COVID-19 pandemic and stay-at-home orders, we hypothesize that ANHPI female students’ risk for SVSH, particularly intimate partner violence (IPV) or dating violence, has increased due to discrimination and isolation. Moreover, we examine the influence of anti-Asian xenophobia due to COVID-19, and visa restrictions applied to the Student and Exchange Visitor Program (SEVP) which could further inhibit Asian international students’ willingness to seek essential SVSH advocacy and support services available on and off campus [32–34].

## Study aims

Due to the increased reports of anti-Asian hate crimes and the concurrent risk of SVSH, an in-depth study is needed to understand the mental health, and help-seeking behaviors of ANHPI female college and university students. However, data on these intersecting problems are almost nonexistent. This lack of data limits our knowledge of the indirect negative consequences of COVID-19, the ways in which sexism and racism interact, and the availability of evidence-based advocacy tools and culturally specific services for ANHPI students who have experienced SVSH.

To address these gaps, the Double Jeopardy Study is launched to collect multidimensional data and increase the policy capacity for ANHPI communities by assessing the experiences of COVID-related SVSH and anti-Asian racism among ANHPI students from the UC, the public university system in California located across 10 locations (Berkeley, Davis, Irvine, Los Angeles, Merced, Riverside, San Diego, San Francisco, Santa Barbara, and Santa Cruz) in the State. The Double Jeopardy Study is initiated in 2021, in response to the increase in anti-Asian racism and SVSH among students at Institutions of Higher Education (IHE) in the U.S. This mixed methods research includes survey data collection, qualitative interviews and participatory-action photovoice research. The methods are informed by intersectional, community-engaged frameworks [35] and guided by trauma-informed practices [36] and survivor-centered principles [37, 38].

Aim 1. Examine associations between physical and mental health (dependent variables) and anti-Asian racism and ethnic microaggressions (independent variables) among approximately 450 ANHPI women who are current or former (within the past year) undergraduate or graduate students across the 10 UC campuses and who experienced SVSH while a UC student. This data is collected through the Double Jeopardy survey questionnaire.

Aim (1.1) Assess exposure to various forms of SVSH – including sexual assault, dating violence, online abuse and stalking, sexual harassment, and forced sex/rape - and compare the change in these exposures before and after the onset of the COVID-19 pandemic (March 11, 2020).

Aim (1.2) Assess exposure to various forms of anti-Asian racism, such as assumed inferiority and exoticization, as well as levels of ethnic microaggressions, including those at environmental and school-levels. Compare the change in these exposures before and after the onset of the COVID-10 pandemic (March 11, 2020).

Aim (1.3) Evaluate self-rated physical and mental health (e.g., depression, anxiety, and PTSD symptoms).

Aim (1.4) Measure the associations between physical and mental health and exposure to anti-Asian racism and ethnic microaggressions.

Aim 2. Examine help-seeking behaviors, perceptions of institutional betrayal (e.g., failure from university administration to prevent or respond supportively to sexual assault), and the use of other community support mechanisms before and after the onset of the COVID-19 pandemic among approximately 450 ANHPI women who are current or recent (within the past year) undergraduate or graduate students across the 10 UC campuses and who experienced SVSH while a UC student. The data is collected through the Double Jeopardy survey questionnaire.

Aim 3. Qualitatively identify barriers and facilitators to seeking advocacy, health and other support services. Investigate perceptions of the impact of COVID-19 on experiences of SVSH and associated help-seeking. Seek recommendations for culturally specific strategies to improve ANHPI SVSH survivors’ access to counseling, healthcare, and legal services.

Aim 4. Use “Trnasmedia-Photovoice” method to give study participants the opportunity to express themselves and share their narratives through photographs, video, and/or other media (i.e., transmedia). Media provided by participants will be used to guide or expand on their interviews, and to disseminate study findings.

### Integrating mixed methods and trauma-informed care in sexual violence research

The application of trauma informed care (TIC) in the research of SVSH has been imperative when working with survivors. Efforts to report incidents and seek out support have been especially difficult for women of color as studies have shown that re-traumatization occurs often when universities and colleges do not provide culturally competent responses [39]. A greater application of TIC in research is associated with a greater likelihood of participants having a positive experience, thus being more willing to participate in the study knowing that they are cared for [40]. Some of these practices include active listening and relationship-building with participants, much of which has been employed in our own study [40].

Numerous studies have sought to understand SVSH among college students, particularly how to prevent it and the help-seeking behaviors of student-survivors [30]. Research has focused on the experience of marginalized and at-risk populations on campuses, such as women of color [41, 42], Lesbian, Gay, Bisexual, Transgender, Queer, and other sexual orientation identities (LGBTQ+) [42–45], individuals involved in Greek life [46–48], international students [49], and athletes [50, 51]. Prior work has discovered multiple barriers to reporting, for example student-survivors do not feel that university or campus police have been helpful and fear of further stigmatization [39]. There is an agreement among literature that a comprehensive approach is necessary when addressing sexual violence on campuses [30, 52–54]. Using the social ecological theory, one study recommended addressing attitudes regarding the continuation of sexual violence, developing programs, and advocating for additional policies to stop the perpetuation of violence as essential in disrupting the status quo [55].

There are a number of relevant studies that have inspired the work of the Double Jeopardy Study. Experiences of violence are dependent on the perspective of the survivor; thus, they cannot be illustrated via solely quantitative methods [56]. The incorporation of mixed methods in SVSH research has allowed researchers to contextualize their quantitative findings with the stories and experiences collected by qualitative methods (i.e., in-depth interviews and Trnasmedia-Photovoice) [56]. This approach allows researchers to capture insights into lived experiences, such as perceptions of ethnic discrimination when interacting with authorities or people in power related to their sexual violence experience.

An integral part of the Double Jeopardy Study is the inclusion of participant stories utilizing photovoice, a powerful participatory research tool for reforming social issues and aiding in the healing process for participants who have faced trauma [57–59]. Photovoice is used in many gender based violence studies to provide participants with the opportunity to discuss issues in a public forum and be involved in the development of a plan to rectify the issue [57, 60]. A previous study sets a successful example by utilizing photovoice to increase the involvement of youth voices in the prevention of sexual and relationship violence [61]. Beyond the collection of qualitative data, photovoice allows victim-survivors to be active participants in the conversation of the issue. Additionally, it can mobilize a community that has not previously existed by collectively contributing to a publicly available project. Through employing the photovoice method, the Double Jeopardy Study project aims to provide a therapeutic tool for participants, and a means for education and activism among college student groups that are often minoritized in the society [62].

## Methods

### Research setting and UC student population demographics

The current study recruited study participants from all 10 UC campuses (e.g., UC Berkeley, UC Davis, UC Irvine, UC Los Angeles, UC Merced, UC Riverside, UC San Diego, UC San Francisco, UC Santa Barbara, UC Santa Cruz). While nine campuses have both undergraduate and graduate programs, UC San Francisco is the only campus with solely a graduate program. At the beginning of the data collection, which took place in Fall 2021, 42,322 undergraduate and 6,745 graduate students across the UC system were identified as Asian women. Additionally, 463 students were identified as NHPI women [31]. Overall student demographic data across 10 UCs in 2021 and 2022 are shown in Appendix 1. We aimed to recruit approximately 450 ANHPI eligible participants and conduct interviews with 50 of them or until we reach saturation. Those who completed an interview were invited to participate in the Trnasmedia-Photovoice.

### Eligibility criteria

Our target population was ANHPI students who have experienced SVSH while attending one of the 10 UC campuses. The eligibility criteria included [1] aged 18 years and over [2], identified as a woman [3], identified with Asian or NHPI ethnicity [4], attended or, graduated in the past 12 months from one of the UC campuses, and [5] experienced SVSH while enrolled as a UC student. We defined these exposures according to the definitions provided in the UC’s Sexual Violence and Sexual Harassment Policy [63] as shown in Table 1.

**Table 1:** Key definitions according to uc’s sexual violence and sexual harassment policy

Sexual Violence	
Sexual Assault - Penetration	Without the consent of the Complainant, penetration, no matter how slight, of the Complainant’s mouth by penis or other genitalia; or the Complainant’s vagina or anus by any body part or object.
Sexual Assault - Contact	Without the consent of the Complainant, intentionally: touching Complainant’s intimate body part (genitals, anus, groin, breast, or buttocks); making the Complainant touch another or themselves on any intimate body part; or touching the Complainant with one’s intimate body part, whether the intimate body part is clothed or unclothed.
Relationship Violence	Physical violence toward the Complainant or a person who has a close relationship with the Complainant, or intentional or reckless physical or non- physical conduct toward the Complainant or someone who has a close relationship with the Complainant that would make a reasonable person in the Complainant’s position fear physical violence toward themselves or toward the person with whom they have the close relationship, that is by a person who is or has been in a spousal, romantic, or intimate relationship with the Complainant, and that is part of a pattern of abusive behavior by the person toward the Complainant.

Sexual Harassment	
Quid Pro Quo	Quid Pro Quo: a person’s submission to unwelcome sexual conduct is implicitly or explicitly made the basis for employment decisions, academic evaluation, grades or advancement, or other decisions affecting participation in a university program or activity.
Hostile Environment	Unwelcome sexual or other sex-based conduct is sufficiently severe, persistent or pervasive that it unreasonably denies, adversely limits, or interferes with a person’s participation in or benefit from the education, employment or other programs or activities of the University, and creates an environment that a reasonable person would find to be intimidating or offensive.

### Study procedures and ethical research

The Double Jeopardy Study employed a sequential approach consisting of three research methodologies: Step 1 involved quantitative online surveys, Step 2 included qualitative in-depth interviews (IDIs), and Step 3 utilized Trnasmedia-Photovoice. Data collection was conducted online following COVID-19 related guidelines from the UCLA Office of Human Research Protection Program (OHRPP). The research protocol was fully reviewed by UCLA’s Institutional Review Board (IRB) and received approval for each step from the UCLA OHRPP (IRB#21–000149 for Steps 1 and 2 and IRB#23–000814 for Step 3). Additionally, we obtained a Certificate of Confidentiality from the National Institute of Health to protect the privacy of research participants by prohibiting the disclosure of identifiable, sensitive information to anyone not connected to the research [64]. Primary data collection took place since September 2021, during which all three methodologies were applied, and data were gathered from eligible participants across the 10 UC campuses.

A major potential risk associated with the Double Jeopardy Study pertains to the sensitivity of the topics being explored. SVSH, racism and other forms of discrimination are stigmatizing, traumatic, and distressing experiences. We recognized that asking participants to recall and share some of these past experiences may exacerbate their trauma and distress. We had multiple safeguards in place to address this issue, such as obtaining informed consent by thoroughly discussing the research team members, study goals, and taking measures to ensure confidentiality and privacy. We took all possible actions to minimize the risks and harm posed to participants. The participants were reminded that their participation in the interview was voluntary and were informed before the interview that they could choose to skip any questions, take a break, or withdraw from the interview at any time if they felt uncomfortable or no longer wished to share their experiences with the research team. After each section of the interview, the participants were informed about the upcoming topic and asked if they would like to continue to the next section. A resource list tailored for college students and ANHPI women was provided to participants following survey and interview completion. For data protection, the computer and storage device were password-protected, and pseudonyms were used for the interviewee’s name. Any identifiable information throughout the transcription and data analysis was removed. Survey participants’ contact information was only collected if they agreed to participate in the interview phase. If the interviewees refused to participate in potential follow-up interviews, all of their contact information was destroyed after the interviews. Furthermore, researchers who conducted IDIs, Trnasmedia-Photovoice, and transcription received additional training in ‘Trauma-Informed Research’ (Appendix 2), designed for the UC Speaks Up research [65] team. To minimize distress from continuous exposure to SVSH content among researchers, pre/post-interview memos (Appendix 3) were utilized to reflect on and discuss challenging aspects of the interviews.

### Step 1. web-based survey

#### Recruitment of the survey participants

We aimed to recruit up to 300 Asian American, 100 Asian international respondents, and 50 NHPI respondents (*N* = 450). We employed a purposive sampling strategy to reach the target population using various methods including, online advertisements on the partnering university organization websites, online flyers, social media postings and direct messaging (e.g., Instagram, Facebook, and Twitter/X), as well as recruitment emails to professors, departments, student advocacy and counseling centers, and student organizations across the 10 UC campuses.

#### Structure of the survey and compensation of participants

Surveys were administered online in English with options for mobile data collection via Qualtrics – an electronic survey platform and were programmed to protect respondent confidentiality and maintain data security. The participants were guided through an online consent process and prompted to “proceed” if they were deemed eligible and consented to take the survey with an option and reminder to stop at any time if they no longer wished to continue. The survey was designed to be completed in 20 to 30 min and those who completed the survey received a $5 online gift card code. Data on demographics like ethnicity, gender identity, sexual orientation, degree program, and international student status, were collected. All the validated survey questionnaires measured each domain of interest, as shown in Table 2..

**Table 2.** Validated survey instruments modules1-6

	Module 1: Exposure & help-seeking	Module 2: Physical and mental health	Module 3: Racial and ethnic microaggression
While attending UC vs. After COVID-19 outbreak	*Component 1*: Sexual harassment (10 Qs) & help-seeking (2 Qs) *Component 2*: Stalking (8 Qs) & help-seeking (2 Qs) *Component 3*: Sexual assault (10 Qs) & help-seeking (2 Qs) *Component 4*: Relationship violence (8 Qs) & help-seeking (2 Qs) ***44 Qs***	Self-rated physical health (2 Qs) Self-rated mental health (2 Qs) 4 ***Qs***	*Component 1*: Assumptions of interiority (16 Qs) *Component 2*: Second-class citizen (18 Qs) *Component 3*: Microinvalidation (18 Qs) *Component 4*: Exoticization (16 Qs) *Component 5*: Environmental microaggression (14 Qs) *Component 6*: School microaggression (10 Qs) ***90 Qs***

	Module 4: Current Mental Health	Module 5: Gendered Racial Microaggressions	Module 6: Institutional Betrayal and Support Questionnaire
Current assessment	*Component 1*: Depression (CESD-R, 18 Qs) *Component 2*: Anxiety (GAD-7, 8 Qs) *Component 3*: PTSD (20 Qs) ***46 Qs***	*Component 1*: Ascription of Submissiveness (9 Qs) *Component 2*: Assumption of Universal Appearance (4 Qs) *Component 3*: Asian Fetishism (5 Qs) *Component 4*: Media Invalidation (4Qs) ***22 Qs***	*Component 1*: Response to survivors *Component 2*: Institutional failures to prevent SVSH *Component 3*: Institutional betrayals related to race and sexual orientation ***26 Qs***

Note. *Qs*: questions

To compare changes that occurred after the onset of COVID-19, defined as after March 11, 2020, Module 1 measured the SVSH exposure and help-seeking behavior before and after the onset of COVID-19 (defined as March 11, 2020). Module 1 contains 10 to 12 questions for each of the four types of SVSH, the help-seeking methods used, and changes before and after the onset of COVID-19 (e.g., more often, less often, same) [66]. Module 2 assessed general self-rated health [67] as well as three domains of mental health before and after the COVID-19. Self-rated health is a widely used indicator of general health status in epidemiologic research [68, 69]. Module 3 measured racial and ethnic microaggression experiences via the Racial and Ethnic Microaggressions Scale (REMS) [70].

To understand the current mental health status of the respondents, three mental health measures were included in Module 4. First, depression was measured by the Patient Health Questionnaire (PHQ-9) which is a self-administered diagnostic instrument for depression with 9 questions about one’s behavioral issues in the past two weeks. The score ranges from 0 to 27 and identifies minimal, mild, moderate, moderately severe, and severe depression (sensitivity of 74% and specificity of 91%) [71, 72]. The Generalized Anxiety Disorder 7-item (GAD-7) is used to assess the level of anxiety in the past two weeks. It is a highly reliable instrument (Cronbach’s alpha = 0.89) [71] that is used to anxiety disorder ranging from minimal anxiety, mild anxiety, moderate anxiety, to severe anxiety (sensitivity of 92% and specificity of 76%) [71, 73, 74]. To better understand, racialized microaggression specific to Asian women, the Gendered Racial Microaggressions Scale for Asian American Women (GRMSAAW) was used in Module 5 [75]. Finally, Module 6 included the Institutional Betrayal and Support Questionnaire (IBSQ), developed by Carly Smith and Jennifer Freyd to evaluate the role of institutional betrayal in cases of sexual assault [76]. The IBQ assesses institutional actions that contribute to or follow an assault, such as creating an environment that minimizes the seriousness of sexual assault or responding inadequately to assault reports [76–78].

#### Statistical analysis of survey data

To understand the distributions and estimate the prevalence of our outcomes of interest (Aims 1–2), we conducted descriptive analyses to assess the frequency distributions of the SVSH variables (Aim 1.1, e.g., sexual harassment, cyber/online/phone harassment, sexual assault, and relationship violence), physical health and mental health variables (Aim 1.2), anti-Asian racism and ethnic microaggression variables (Aim 1.3) and help-seeking behaviors, perceptions of institutional betrayal and the utilization of other community support mechanisms (Aim 2). To estimate the differences in the Aim 1–2 outcomes before and after the onset of COVID-19, we performed paired two-sample t-tests at a significance level of α = 0.05. Additionally, multivariate regression analyses were performed as the last step to examine the associations between potential predictors of certain help-seeking behaviors or health outcomes depending on the type of exposure to violence. We stratified the study samples by Asian ethnicities (e.g., Chinese, Korean, Filipinos, etc.) and Asian regional groups (e.g., Southeast Asian, East Asian, South Asian, etc.) to conduct an analysis of the experiences of various sub-Asian groups. We limited this stratification approach if the cell sizes become too small (i.e., if there are too few subjects in any one group). Similarly, we disaggregated the data by Asian American, Asian International, and NHPI status when statistically possible. All statistical analyses were conducted using STATA.

### Step 2. in-depth interviews

#### Recruitment of IDI participants

After the participants completed the survey at Step 1, they were asked if they were willing to be contacted for a follow-up qualitative interview. The participants who expressed a willingness to participate in the qualitative interview were then contacted via email to schedule their interviews with our team of researchers. Informed consent was then received after each participant was informed of the measures in place to ensure their privacy and their ability to terminate the interview process at any time.

#### Structure of IDIs and compensation of participants

The semi-structured interview was designed to explore: [1] the barriers that prevented student-survivors from seeking SVSH- and health-related services; [2] the factors contributing to student survivors’ help-seeking behaviors; [3] the perceived impact of COVID-19 on SVSH and help-seeking; and [4] culturally-appropriate strategies and practices to facilitate help-seeking among Asian American, Asian International, and NHPI student survivors to access counseling, healthcare, and legal services (Appendix 4 for the interview guide). The semi-structured interview guide allowed for probing and expansion of topics as appropriate, ensuring a comprehensive exploration of the research questions. To ensure the comfort and safety of the participants, the interviews were offered in English, Chinese, or Korean, depending on the participant’s preference. These languages were provided based on the basis of the high percentage of Asian international students from China and South Korea attending UCs. The participants were informed that other Asian languages would also be made available upon request.

The interviewers received training in trauma-responsive approaches and employed various techniques throughout the interviews to prioritize the participants’ well-being. The participants were informed at the beginning of the interview that they could terminate the interview at any point, had the freedom to choose a pseudonym at the beginning of the interview, and could request a break from questions or to skip any questions at any time throughout the interviews. During each section of the interview guide, interviewers were prompted to start by sharing the topics of each cluster of questions and providing content warnings prior to the questions as needed. Among our research team, constant check-ins were made throughout the data collection. Pre/post-interview memos were utilized as part of the data collection (Appendix 3) to allow the researcher to reflect on the interview experience and facilitate in-depth research team discussions.

All interviews were audio-recorded with participants’ consent for transcription purposes. The participants received a $50 gift card to compensate for their time. The e-gift card was emailed to them after they were interviewed along with a list of SVSH advocacy services available online, at the UC, or near their college campuses.

#### Qualitative analysis

The qualitative data analysis was conducted based on a grounded theory-guided thematic analysis technique [79, 80]. Each interview was transcribed using the online transcription service called Trint. Trint uses the Amazon AWS server, and the transcription content was stored in encrypted AWS S3 buckets. Once the transcripts were available, we performed initial coding to identify key themes and patterns within the data. This coding process involved line by line open coding for 16 randomly selected transcripts to identify and label concepts. Memoing was employed throughout the analysis to document the researcher’s reflections, biases, and insights, which facilitated a more nuanced interpretation of the data. These memos provided valuable context and aided in the development of a conceptual framework. After initial coding, we organized the codes into categories, considering different dimensions to allow for comparisons of incidents across all qualitative data. We, then, applied the initial codes to the remaining transcripts and continued to evolve and refine the coding tree as we analyzed further transcripts. This comparative analysis enabled us to refine the emerging theoretical concepts and generate a grounded understanding of the participants’ experiences.

The goal was to achieve saturation, wherein further coding and analysis did not yield any new themes. This process led to the development of a theoretical explanatory model that describes a spectrum of experiences of SVSH among the ANHPI student population, including their help-seeking behaviors and potential barriers to accessing resources.

### Step 3. transmedia-photovoice

We developed the Transmedia-Photovoice by adapting the Photovoice method. Photovoice was developed by Caroline C. Wang and Mary Ann Burris in the early 1990s as part of a participatory action research (PAR) approach [81–84]. Photovoice allowed people who do not usually have a say in the decisions that affect their daily lives to deepen their understanding of an issue [60, 85, 86]. Photovoice was a participatory research method that engaged and empowered participants to tell their own personal, lived stories through photography and group dialogue. Photovoice provided the participant with multiple media platforms (e.g., drawings, films, mobile apps) to use to expand their narrative across time. Photovoice storytelling approaches were found to provide a more culturally competent way for marginalized individuals to both express themselves and deepen their understanding of important, often traumatic, issues or concerns [60, 81–83, 86]. The goal of Photovoice was to support the self-empowerment of participants by providing them with the opportunity to express their experiences and “speak” through photographs about issues that bother them, provide an avenue to connect with others in their community, and advocate for change [81, 82, 84]. Photovoice allowed people in a community to express the concerns and issues that were most important and relevant to them. Because “a picture is worth a thousand words”, it was a powerful way to help others understand and connect with the issues. It could both effectively support ANHPI student victim-survivors to support their healing processes and raise public awareness of the multiple forms of violence and discrimination against the ANHPI student population across California and the U.S.

#### Recruitment of transmedia-photovoice participants

All interview participants were invited to take part in the Transmedia-Photovoice. Those who agreed to participate in the Transmedia-Photovoice project were scheduled for Zoom meetings with a member of our research team to discuss the study’s goals and process. Participants signed both an informed consent form and a media release form, which detailed the project’s objectives, intended use of the submitted photography and multimedia pieces, and confidentiality issues. Over a two- to four-week period, participants were asked to capture photographs or create other forms of visual media. Each week, the participants communicated with our researchers about the pictures they took and what they represented for them. The participants discussed with our research team their creative process, the photographs/transmedia they created, and their narratives to reflect the spectrum of violence, coping, and physical and mental health they wanted to convey in the artwork. Finally, study participants were invited to create an exhibit to give public voice to the present and educate the community about ANHPI students’ experiences.

#### Structure of transmedia-photovoice and the compensation of participants

Three key steps were involved in Transmedia-Photovoice:

1. Collaboratively planning a project: Involved thinking about who needs to be involved, how to recruit participants and creating a timeline for the entire process.
2. Supporting the execution of individual project: Included introducing Transmedia-Photovoice to each participant, reviewing ethical guidelines, providing technical support for transmedia artwork creation, and discussing artworks created by participants. Each participant created artwork that they felt answered the research question relating both to their personal experience and aspects of their community’s strengths, struggles and collective experiences. In the last meeting, participants shared the meanings and stories behind the artwork and discussed themes that emerged.
3. Exhibiting artworks and creating community action: Involved the whole group of participants, research team, and community members deciding how they wanted to share their pictures and stories with others in the community and beyond and thinking about who they want to reach (target audience).

The participants were given a $50 gift code at the end of each meeting/session with the research team for the Transmedia-Photovoice project. They were invited to two online meetings and one in-person exhibition event. Each participant was eligible to be compensated up to $150 during the Transmedia-Photovoice. Each participant was permitted to submit multiple formats of Transmedia-Photovoice work (Appendix 5 for examples of the submitted works).

#### Guided questions for reflection

We used Caroline Wang’s structured technique of photo selection and guided dialogue [81]. Each participant selected one to five favorite Transmedia-Photovoice pieces. The dialogue around the selected pieces was guided by technique called, “SHOWED” [81]. The letters of this acronym each corresponded to a question and the series of questions prompted the participants to critically analyze the content of their Transmedia-Photovoice pieces. The participants then codified their issues, themes and theories emerging from the Transmedia-Photovoice pieces [82].

- S: What do we See here?
- H: What is really Happening here?
- O: How does this relate to Our lives?
- WE: Why does this situation, concern or strength Exist?
- D: What can you or others Do about it?”

The participants then interpreted and analyzed the photographs by responding to in-depth questions. The importance of dialogue was emphasized in order to help the participants gain a clearer sense of the stories they wanted to accompany their photographs [87].

- What did the photographs mean to you?
- What was the relationship between the content of the photographs and themes related to sexual violence, microaggression/discrimination/racism against AANHPI women, COVID-19, and physical and mental health?
- How did you see the photographs as reflecting issues that are salient to you?

#### Community exhibition and collaboration

**Fig. 1:**
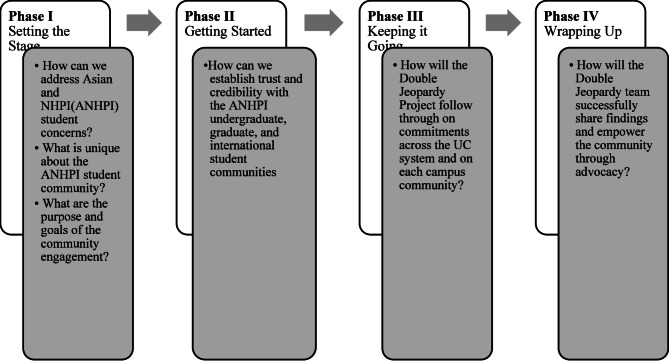
Phases of double jeopardy study community engagement plan

Collaborating with ANHPI students and local community members was the foundation of the Double Jeopardy Study’s efforts to prevent violence, racism and discrimination, improve health, and promote social justice. We collaborated with ANHPI student groups as well as SVSH advocacy groups across UCs and universities and colleges in Los Angeles to host community exhibitions and create spaces for discussion (Appendix 6). Moving forward, we continue to build on our existing partnerships and coalitions, and to find, create and share resources in order to influence the UC system and foster changes in policies, programs, and practices. To provide a structured framework, we followed the Community Engagement Planning Tool for Public Health Work, developed by the U.S. Centers for Disease Control and Prevention, as a guide for implementation of community engagement with students and other groups throughout the study period [88]. This approach involves four phases to community engagement, including: Setting the Stage, Getting Started, Keeping it Going, and Wrapping Up as shown in Fig. 1 [88].

## Discussion

To our knowledge, the Double Jeopardy Study was the first to integrate rigorous public health research with Transmedia-Photovoice methods, aiming to increase awareness and advocate for the health, rights, and safety of ANHPI students who have experienced SVSH and racism at higher education institutions who have experienced SVSH and racism. The findings and recommendations were planned for dissemination across the University of California system, within local communities, throughout California, in collaboration with community members, ANHPI student groups, and local partners such as the Asian Pacific Institute on Gender-Based Violence, the Center for the Pacific Asian Family, and the Asian Pacific Student Coalition. We organized panel discussions and university community exhibitions to translate academic findings into practical lessons that resonated with the local community and contributed to developing culturally competent approaches. These efforts were designed to empower ANHPI individuals to express themselves, deepen community members’ and stakeholders’ understanding of critical issues, and enhance university initiatives in SVSH prevention and response structures tailored for students’ needs.

### Strengths and limitations

The study employed a mixed-methods approach, combining quantitative surveys, qualitative interviews, and participatory photovoice methods, which provided a comprehensive understanding of ANHPI students’ experiences. This approach allowed for a rich and nuanced exploration of ANHPI students’ experiences of SVSH victimization, gendered and racialized microaggressions, and intrapersonal, interpersonal, and social system navigation for coping and healing. The research was guided by a trauma-informed framework, ensuring that participant-centered and ethically sound processes were followed, such as self-reflective memo-ing, reflective group discussions, referrals to university and community resources, frequent content warnings, and offering participants the option to end the interview at any time without a penalty. This study prioritized the well-being of participants and demonstrated cultural competence by employing strategies such as providing language options and addressing cultural stigmas, with researchers from diverse backgrounds who could relate to the participants’ own experiences, thereby enhancing the relevance and accuracy of the findings. The interviews conducted in Chinese and Korean were examples of this strength. Additionally, the study was led by university students who were familiar with ANHPI student organizations and advocacy groups for SVSH across the UC, fostering trust and access, particularly among groups typically underrepresented in SVSH research. As a result, 39% of the participants were international students, a demographic often underrepresented in other studies of SVSH.

However, the study had several limitations. Its focus on ANHPI female UC students who experienced SVSH may limit the generalizability of findings to other university campuses, as the types and quality of services available at UCs may differ elsewhere. Additionally, self-reported data on sexual violence and discrimination introduces potential recall and social desirability biases, particularly when comparing pre- and post-COVID-19 experiences. The interviews were used to contextualize the quantitative data, but this method has its own limitations. Recruitment was primarily online, potentially excluding individuals with limited internet access or those who were less engaged with digital platforms. In-person recruitment was limited to UCLA and UCSD, whereas mailed surveys were sent to UC Berkeley, contributing to sample imbalances across campuses. Furthermore, recruiting NHPI students was particularly difficult, leading to their underrepresentation. This highlights the need for a separate, focused study on the NHPI and other Native American student populations to better capture their unique experiences.

## Supplementary Information

Supplementary Material 1

Supplementary Material 2

Supplementary Material 3

Supplementary Material 4

Supplementary Material 5

Supplementary Material 6

## Data Availability

No datasets were generated or analysed during the current study.

## Abbreviations

SVSH: Sexual Violence and Sexual Harassment
PTSD: Post-Traumatic Stress Disorder
COVID-19: Coronavirus disease 2019
U.S.: United States
ANHPI: Asian, Native Hawaiian, and Pacific Islander
UC: University of California
IPV: Intimate Partner Violence
SEVP: Student and Exchange Visitor Program
IHE: Institutions of Higher Education
TIC: Trauma Informed Care
LGBTQ+: Lesbian, Gay, Bisexual, Transgender, Queer, and other sexual orientation identities
IDIs: In-depth Interviews
OHRPP: Office of Human Research Protection Program
IRB: Institutional Review Board
REMS: Racial and Ethnic Microaggressions Scale
PHQ-9: Patient Health Questionnaire
GAD-7: Generalized Anxiety Disorder 7-item
GRMSAAW: Gendered Racial Microaggressions Scale for Asian American Women
IBSQ: Institutional Betrayal and Support Questionnaire
PAR: Participatory Action Research
STEM: Science, Technology, Engineering, and Mathematics

## Footnotes

1 We use the term ‘victim-survivor’ to acknowledge both the harm and trauma experienced as well as the resilience demonstrated. This term provides a more inclusive and nuanced understanding of individuals navigating their path toward healing, respecting their personal identities and recognizing that recovery is a complex and ongoing process [26, 27].

## References

[1] Cantor D, Fisher B, Chibnall S, Townsend R, Lee H, Bruce C, et al. Report on the AAU campus climate survey on sexual assault and sexual misconduct. [Internet]: Association of American Universities. 2015. https://www.aau.edu/sites/default/files/AAU-Files/Key-Issues/Campus-Safety/AAU-Campus-Climate-Survey-FINAL-10-20-17.pdf.

[2] Dills J, Fowler D, Payne G. Sexual violence on campus: strategies for prevention. Atlanta, GA: National Center for Injury Prevention and Control, Centers for Disease Control and Prevention; 2016. Available from: https://stacks.cdc.gov/view/cdc/43899. Cited 2024 Jul 24.

[3] Carlson BE, McNutt LA, Choi DY. Childhood and adult abuse among women in primary health care: effects on mental health. J Interpers Violence. 2003;18(8):924–41. 19768893 10.1177/0886260503253882

[4] Straight ES, Harper FWK, Arias I. The impact of partner psychological abuse on health behaviors and health status in college women. J Interpers Violence. 2003;18(9):1035–54. 19771708 10.1177/0886260503254512

[5] Kendra R, Bell KM, Guimond JM. The impact of child abuse history, PTSD symptoms, and anger arousal on dating violence perpetration among college women. J Fam Violence. 2012;27(3):165–75.

[6] Dating Violence Among College Students. The Risk and Protective Factors - Catherine Kaukinen, 2014. Available from: https://journals.sagepub.com/doi/10.1177/1524838014521321. Cited 2024 Jul 24. 10.1177/1524838014521321 24499962

[7] Victimization DV, Satisfaction R, Problems MH. and Acceptability of Violence: A Comparison of Men and Women | Journal of Family Violence. . Available from: https://link.springer.com/article/10.1007/s10896-007-9092-0. Cited 2024 Jul 24.

[8] Eisenberg ME, Lust K, Mathiason MA, Porta CM. Sexual assault, sexual orientation, and reporting among college students. J Interpers Violence. 2021;36(1–2):62–82. 29294876 10.1177/0886260517726414

[9] Potter S, Howard R, Murphy S, Moynihan MM. Long-term impacts of college sexual assaults on women survivors’ educational and career attainments. J Am Coll Health. 2018;66(6):496–507. 29447618 10.1080/07448481.2018.1440574

[10] Santaularia J, Johnson M, Hart L, Haskett L, Welsh E, Faseru B. Relationships between sexual violence and chronic disease: a cross-sectional study. BMC Public Health. 2014;14(1):1286. 25516229 10.1186/1471-2458-14-1286 PMC4302144

[11] Racism & Sexual Violence. What’s the Connection? | PCAR. Available from: https://pcar.org/resource/racism-sexual-violence-whats-connection-1. Cited 2024 Jul 24.

[12] Fethi I, Daigneault I, Bergeron M, Hébert M, Lavoie F. Campus sexual violence: a comparison of international and domestic students. J Int Stud. 2023;13(1):1–21.

[13] Scott CV, Singh AA, Harris JC. The intersections of lived oppression and resilience: sexual violence prevention for women of color on college campuses. In: Harris JC, Linder C, eds. Intersections of identity and sexual violence on campus. Routledge; 2017:119–139.

[14] Wu C, Qian Y, Wilkes R. Anti-Asian discrimination and the Asian-white mental health gap during COVID-19. Ethn Racial Stud. 2021;44(5):819–835. 10.1080/01419870.2020.1851739.

[15] Gover AR, Harper SB, Langton L. Anti-Asian hate crime during the COVID-19 pandemic: exploring the reproduction of inequality. Am J Crim Justice. 2020;45(4):647–67. 32837171 10.1007/s12103-020-09545-1 PMC7364747

[16] Wang SC, Santos BMC. Go back to China with your (expletive) virus: a revelatory case study of anti-Asian racism during COVID-19. Asian Am J Psychol. 2022;13(3):220–33.

[17] Haft SL, Zhou Q. An outbreak of xenophobia: perceived discrimination and anxiety in Chinese American college students before and during the COVID-19 pandemic. Int J Psychol. 2021;56(4):522–31. 33426695 10.1002/ijop.12740 PMC7962181

[18] Chiang PP. Anti-Asian racism, responses, and the impact on Asian americans’ lives: A social-ecological perspective. In: Ryan JM, ed. COVID-19. Routledge; 2020:215–229.

[19] Hahm HC, Xavier Hall CD, Garcia KT, Cavallino A, Ha Y, Cozier YC, et al. Experiences of COVID-19-related anti-Asian discrimination and affective reactions in a multiple race sample of U.S. young adults. BMC Public Health. 2021;21(1):1563. 34407792 10.1186/s12889-021-11559-1 PMC8371291

[20] Lee S, Waters SF. Asians and Asian Americans’ experiences of racial discrimination during the COVID-19 pandemic: impacts on health outcomes and the buffering role of social support. Stigma Health. 2021;6(1):70–8.

[21] Han S, Riddell JR, Piquero AR. Anti-Asian American hate crimes spike during the early stages of the COVID-19 pandemic. J Interpers Violence. 2023;38(3–4):3513–33. 35657278 10.1177/08862605221107056 PMC9168424

[22] Yi J, La R, Lee BA, Saw A. Internalization of the model minority myth and sociodemographic factors shaping Asians/Asian Americans’ experiences of discrimination during COVID-19. Am J Community Psychol. 2022. 10.1002/ajcp.12635. 36440675 10.1002/ajcp.12635 PMC9877560

[23] Harris JC, Linder C. Intersections of identity and sexual violence on campus: centering minoritized students’ experiences / edited by Jessica C. Harris and Chris Linder, foreword by Wagatwe Wanjuki. First edition. Sterling, Virginia: Stylus Publishing, LLC; 2017.

[24] Armstrong EA, Gleckman-Krut M, Johnson L. Silence, Power, and inequality: an intersectional approach to sexual violence. Annu Rev Sociol. 2018;44(44, 2018):99–122.

[25] Crenshaw K. Mapping the margins: intersectionality, identity politics, and violence against women of color. Stanford Law Rev. 1991;43(6):1241–99.

[26] Boyle KM, Rogers KB. Beyond the rape victim–survivor binary: how race, gender, and identity processes interact to shape distress. Sociol Forum. 2020;35(2):323–45.

[27] Papendick M, Bohner G. Passive victim – strong survivor? Perceived meaning of labels applied to women who were raped. PLoS One. 2017;12(5):e0177550. 28493976 10.1371/journal.pone.0177550 PMC5426776

[28] Espinosa DM. Not your submissive China doll: counseling Asian American female survivors of sexual assault at the intersection of racialized sexism. Asian Am J Psychol. 2023;14(3):284–96.

[29] Lai J, Park E, Amabile CJ, Boyce SC, Fielding-Miller R, Swendeman D. “They Don’t See Us”: Asian Students’ Perceptions of Sexual Violence and Sexual Harassment on Three California Public University Campuses. J Interpers Violence. 2024;39(15–16):3619–50. 38470066 10.1177/08862605241235912 PMC11283745

[30] Bloom BE, Park E, Swendeman D, Oaks L, Sumstine S, Amabile C, et al. Opening the black box: student-generated solutions to improve sexual violence response and prevention efforts for undergraduates on college campuses. Violence Against Women. 2022;28(14):3554–87. 35040708 10.1177/10778012211068063 PMC11389659

[31] University of California. Fall enrollment at a glance. Fall enrollment at a glance. Available from: https://www.universityofcalifornia.edu/about-us/information-center/fall-enrollment-glance. Cited 2024 Jul 24.

[32] Liu H, Wong YJ, Mitts NG, Li PFJ, Cheng J. A phenomenological study of East Asian international students’ experience of counseling. Int J Adv Couns. 2020;42(3):269–91.

[33] Ko SJS, Acculturation on Psychological Wellbeing of East Asian International. Examining the Impact of Discrimination, Shame, and Students [Ph.D.]. [United States -- Colorado]: University of Denver; 2022. Available from: https://www.proquest.com/docview/2718672494/abstract/67E26C94FDF84C9BPQ/1. Cited 2024 Jul 27.

[34] Anandavalli S, Borders LD, Kniffin LE. Because here, white is right: mental health experiences of international graduate students of color from a critical race perspective. Int J Adv Couns. 2021;43(3):283–301. 34054168 10.1007/s10447-021-09437-x PMC8142606

[35] McCauley HL, Campbell R, Buchanan NT, Moylan CA. Advancing theory, methods, and dissemination in sexual violence research to build a more equitable future: an intersectional, community-engaged approach. Violence Against Women. 2019;25(16):1906–31. 31530103 10.1177/1077801219875823

[36] Campbell R, Goodman-Williams R, Javorka M. A trauma-informed approach to sexual violence research ethics and open science. J Interpers Violence. 2019;34(23–24):4765–93. 31514606 10.1177/0886260519871530

[37] Ghanbarpour S, Palotai A, Kim ME, Aguilar A, Flores J, Hodson A, et al. An exploratory framework for community-led research to address intimate partner violence: a case study of the survivor-centered advocacy project. J Fam Violence. 2018;33(8):521–35.

[38] Luo Y, Chen H, Hecht L, Fan S, Lai YH. Experiences of East Asian International Counseling Students during the COVID-19 Pandemic. | Journal of Asia Pacific Counseling | EBSCOhost. Vol. 13. 2023;15. Available from: https://openurl.ebsco.com/contentitem/doi:10.18401%2F2023.13.1.2?sid=ebsco:plink:crawler&id=ebsco:doi:10.18401%2F2023.13.1.2. Cited 2024 Jul 27.

[39] Burton CW, Guidry JD. Reporting intimate partner violence and sexual assault: a mixed methods study of concerns and considerations among college women of color. J Transcult Nurs. 2021;32(4):370–81. 32666892 10.1177/1043659620941583

[40] Anderson KM, Karris MY, DeSoto AF, Carr SG, Stockman JK. Engagement of sexual violence survivors in research: Trauma-informed research in the THRIVE study. Violence Against Women. 2023;29(11):2239–65. 36148910 10.1177/10778012221125501 PMC10387722

[41] Raj A, Johns N, Jose R. Racial/ethnic disparities in sexual harassment in the United States, 2018. J Interpers Violence. 2021;36(15–16):NP8268-89. 30973044 10.1177/0886260519842171

[42] Ussher JM, Hawkey A, Perz J, Liamputtong P, Sekar J, Marjadi B, et al. Crossing boundaries and fetishization: experiences of sexual violence for trans women of color. J Interpers Violence. 2022;37(5–6):NP3552–84. 32783523 10.1177/0886260520949149

[43] Bloom BE, Kieu TK, Wagman JA, Ulloa EC, Reed E. Responsiveness of sex education to the needs of LGBTQ + undergraduate students and its influence on sexual violence and harassment experiences. Am J Sex Educ. 2022;17(3):368–99.

[44] Martin-Storey A, Paquette G, Bergeron M, Dion J, Daigneault I, Hébert M, et al. Sexual violence on campus: differences across gender and sexual minority status. J Adolesc Health. 2018;62(6):701–7. 29573883 10.1016/j.jadohealth.2017.12.013

[45] McMahon S, Seabrook RC. Reasons for nondisclosure of campus sexual violence by sexual and racial/ethnic minority women. J Stud Aff Res Pract. 2020;57(4):417–31.

[46] Marcantonio TL, Hunt ME, Schisler E. Assessing sorority women’s perceptions of barriers to reporting sexual assaults that occur within college campus Greek organizations. J Child Sex Abuse. 2023;32(3):359–78. 10.1080/10538712.2023.2189195 36912376

[47] Mazar LA, Kirkner A. Fraternities and campus sexual violence: risk, protection, and prevention. Violence and Gender. 2016;3(3):132–8.

[48] Armstrong EA, Hamilton L, Sweeney B. Sexual assault on campus: a multilevel, integrative approach to party rape. Soc Probl. 2006;53(4):483–99.

[49] Bonistall Postel EJ. Violence against international students: A critical gap in the literature. Trauma Violence Abuse. 2020;21(1):71–82. 29333995 10.1177/1524838017742385

[50] Adhia A, Ellyson AM, Kroshus E. Prevalence and formal reporting of sexual violence among undergraduate student-athletes: a multi-state study. J Interpers Violence. 2023;38(1–2):418–42. 10.1177/08862605221081936 35475767

[51] Carey DS, Sumstine S, Amabile C, Helvink H, Sorin CR, Swendeman D, et al. Student-Athletes’, Coaches’, and administrators’ perspectives of sexual violence prevention on three campuses with National collegiate athletic association division I and II athletic programs. J Interpers Violence. 2022;37(13-14). 10.1177/08862605211067018. 10.1177/08862605211067018 PMC11321757 35259318

[52] Steele B, Martin M, Sciarra A, Melendez-Torres GJ, Degli Esposti M, Humphreys DK. The prevalence of sexual assault among higher education students: a systematic review with meta-analyses. Trauma Violence Abuse. 2024;25(3):1885–98. 37728132 10.1177/15248380231196119 PMC11155219

[53] Basile KC. A comprehensive approach to sexual violence prevention. N Engl J Med. 2015;372(24):2350–2. 26061841 10.1056/NEJMe1503952 PMC4699676

[54] McMahon S, Steiner JJ, Snyder S, Banyard VL. Comprehensive prevention of campus sexual violence: expanding who is invited to the table. Trauma Violence Abuse. 2021;22(4):843–55. 31690226 10.1177/1524838019883275

[55] Bonar EE, DeGue S, Abbey A, Coker AL, Lindquist CH, McCauley HL, et al. Prevention of sexual violence among college students: current challenges and future directions. J Am Coll Health. 2022;70(2):575–88. 32407244 10.1080/07448481.2020.1757681 PMC7666108

[56] Testa M, Livingston JA, VanZile-Tamsen C. Advancing the study of violence against women using mixed methods: integrating qualitative methods into a quantitative research program. Violence Against Women. 2011;17(2):236–50. 21307032 10.1177/1077801210397744 PMC3053530

[57] Christensen MC. Using photovoice to address Gender-Based violence: A qualitative systematic review. Trauma Violence Abuse. 2019;20(4):484–97. 29333971 10.1177/1524838017717746

[58] To PDN, Huynh J, Wu JTC, Vo Dang T, Lee C, Tanjasiri SP. Through our eyes, hear our stories: a virtual photovoice project to document and archive Asian American and Pacific Islander community experiences during COVID-19. Health Promot Pract. 2022;23(2):289–95. 35285319 10.1177/15248399211060777

[59] Banerjee AT, Islam S, Khan A, Hussain N, Ascencio E, Hafleen N. Beyond the body: using photovoice to explore social determinants of diabetes with South Asian adolescents in the Peel region of Ontario, Canada. Can J Diabetes. 2024;48(2):97–e1043. 37952645 10.1016/j.jcjd.2023.11.002

[60] Christensen MC, Caswell C, Yilmazli Trout I, Tose S. Engaging photovoice to complement on sexual assault climate research: a literature review, case study, and recommendations. J Community Psychol. 2021;49(6):1692–706. 34125954 10.1002/jcop.22622

[61] Banyard VL, Ward S, Cohn ES, Plante EG, Moorhead C, Walsh W. Unwanted sexual contact on campus: a comparison of women’s and men’s experiences. Violence Vict. 2007;22(1):52–70. 17390563 10.1891/vv-v22i1a004

[62] Frohmann L. The framing safety project: photographs and narratives by battered women. Violence Against Women. 2005;11(11):1396–419. 16204731 10.1177/1077801205280271

[63] University of California Title IX Office. University of California – Sexual Violence and Sexual Harassment. University of California. 2022. Available from: https://sexualharassment.ucla.edu/file/1aba2404-7ff1-4330-8919-f50f5a5e5eb5

[64] Check DK, Wolf LE, Dame LA, Beskow LM. Certificates of confidentiality and informed consent: perspectives of IRB chairs and institutional legal counsel. IRB. 2014;36(1):1–8. 24649737 PMC4076050

[65] Wagman JA, Amabile C, Sumstine S, Park E, Boyce S, Silverman J, et al. Student, staff, and faculty perspectives on intimate partner and sexual violence on 3 public university campuses: protocol for the UC speaks up study and preliminary results. JMIR Res Protoc. 2022;11(4):e31189. 35380114 10.2196/31189 PMC9019617

[66] Center on Gender Equity and Health. EMERGE COVID-19 and Gender Questions Partner Violence, Sexual Exploitation and Bystander Behavior. 2020. Available from: https://emerge.ucsd.edu/wp-content/uploads/2020/07/emerge-covid-and-gender-questions-partner-violence-sexual-exploitation-and-bystander-behavior.pdf

[67] Center on Gender Equity and Health. EMERGE COVID-19 and Gender Questions Physical and Mental Health. 2020. Available from: https://emerge.ucsd.edu/wp-content/uploads/2020/07/emerge-covid-and-gender-questions-physical-and-mental-health.pdf

[68] Fayers PM, Sprangers MA. Understanding self-rated health. Lancet. 2002;359(9302):187–8. 11812551 10.1016/S0140-6736(02)07466-4

[69] Zajacova A, Dowd JB. Reliability of self-rated health in US adults. Am J Epidemiol. 2011;174(8):977–83. 21890836 10.1093/aje/kwr204 PMC3218632

[70] Nadal KL. The Racial and ethnic microaggressions scale (REMS): Construction, reliability, and validity. J Couns Psychol. 2011;58(4):470–80. 21875180 10.1037/a0025193

[71] Kroenke K, Spitzer RL, Williams JBW. The PHQ-9. J Gen Intern Med. 2001;16(9):606–13. 11556941 10.1046/j.1525-1497.2001.016009606.x PMC1495268

[72] Arroll B, Goodyear-Smith F, Crengle S, Gunn J, Kerse N, Fishman T, et al. Validation of PHQ-2 and PHQ-9 to screen for major depression in the primary care population. Ann Fam Med. 2010;8(4):348–53. 20644190 10.1370/afm.1139 PMC2906530

[73] Spitzer RL, Kroenke K, Williams JBW, Löwe B. A brief measure for assessing generalized anxiety disorder: the GAD-7. Arch Intern Med. 2006;166(10):1092–7. 16717171 10.1001/archinte.166.10.1092

[74] Plummer F, Manea L, Trepel D, McMillan D. Screening for anxiety disorders with the GAD-7 and GAD-2: a systematic review and diagnostic metaanalysis. Gen Hosp Psychiatry. 2016;39:24–31. 26719105 10.1016/j.genhosppsych.2015.11.005

[75] Keum BT, Brady JL, Sharma R, Lu Y, Kim YH, Thai CJ. Gendered racial microaggressions scale for Asian American women: development and initial validation. J Couns Psychol. 2018;65(5):571–85. 30058827 10.1037/cou0000305

[76] Smith CP, Freyd JJ. Institutional betrayal. Am Psychol. 2014;69(6):575–87. 25197837 10.1037/a0037564

[77] Smith CP, Freyd JJ. Dangerous safe havens: institutional betrayal exacerbates sexual trauma. J Trauma Stress. 2013;26(1):119–24. 23417879 10.1002/jts.21778

[78] Smith CP, Freyd JJ. Insult, then injury: interpersonal and institutional betrayal linked to health and dissociation. J Aggress Maltreat Trauma. 2017;26(10):1117–31.

[79] Braun V, Clarke V. Using thematic analysis in psychology. Qual Res Psychol. 2006;3(2):77–101.

[80] Charmaz K. Constructing grounded theory: A practical guide through qualitative analysis. 2nd ed. SAGE Publications; 2014.

[81] Wang CC. Photovoice. A participatory action research strategy applied to women’s health. J Womens Health. 1999;8(2):185–92. 10100132 10.1089/jwh.1999.8.185

[82] Wang C, Burris MA. Photovoice. Concept, methodology, and use for participatory needs assessment. Health Educ Behav. 1997;24(3):369–87. 9158980 10.1177/109019819702400309

[83] Suprapto N, Sunarti T, Suliyanah, Wulandari D, Hidayaatullaah HN, Adam AS, et al. A systematic review of photovoice as participatory action research strategies. Int J Eval Res Educ. 2020;9(3):675–83.

[84] Budig K, Diez J, Conde P, Sastre M, Hernán M, Franco M. Photovoice and empowerment: evaluating the transformative potential of a participatory action research project. BMC Public Health. 2018;18(1):432. 29609576 10.1186/s12889-018-5335-7 PMC5879794

[85] Sinko L, Saint Arnault D. Photo‐experiencing and reflective listening: a trauma‐informed photo‐elicitation method to explore day‐to‐day health experiences. Public Health Nurs. 2021;38(4):661–70. 33813744 10.1111/phn.12904

[86] Rolbiecki A, Anderson K, Teti M, Albright DL. Waiting for the cold to end: using photovoice as a narrative intervention for survivors of sexual assault. Traumatology. 2016;22(4):242–8.

[87] Mcintyre A. Through the eyes of women: photovoice and participatory research as tools for reimagining place. Gend Place Cult. 2003;10(1):47–66.

[88] Agency for Toxic Substances and Disease Registry. Community Engagement Planning Tool for Public Health Work. Available from: https://www.atsdr.cdc.gov/ceplaybook/docs/ce-planning-tool-form-508.pdf

