# Supplementary material for: Double jeopardy study protocol: mixed-methods study to understand ANHPI college students at the intersection of sexual violence and anti-Asian racism after COVID-19
Source: BMC Public Health. 2025 Dec 22;25:4275. doi: 10.1186/s12889-025-25533-8 (PMC12723919; doi:10.1186/s12889-025-25533-8)
Supplement: Supplementary file 1 — Supplementary Material 1 [file 12889_2025_25533_MOESM1_ESM.docx]

## Appendix 1. University of California Student Demographics in 2021 and 2022

Table 1. Student Demographics in University of California in 2021 and 2022^[[1]](#footnote-1)^

| **Year** | **2021** |  | **2022** |  |
| --- | --- | --- | --- | --- |
| **Undergraduate** | **N** | **%** | **N** | **%** |
| African American | 9,886 | 4.3% | 10,124 | 4.4% |
| American Indian | 1,025 | 0.4% | 1,149 | 0.5% |
| Asian | 79,999 | 34.7% | 82,271 | 35.7% |
| Domestic unknown | 6,000 | 2.6% | 5,826 | 2.5% |
| Hispanic/Latino(a) | 57,944 | 25.1% | 58,404 | 25.3% |
| International | 26,962 | 11.7% | 24,496 | 10.6% |
| Pacific Islander | 575 | 0.2% | 533 | 0.2% |
| White | 48,138 | 20.9% | 47,604 | 20.7% |
| **Sub-total** | **230,529** |  | **230,407** |  |
| **Graduate** | **N** | **%** | **N** | **%** |
| African American | 3,196 | 5.0% | 3,253 | 5.1% |
| American Indian | 442 | 0.7% | 415 | 0.7% |
| Asian | 12,523 | 19.6% | 12,508 | 19.6% |
| Domestic unknown | 2,545 | 4.0% | 2,472 | 3.9% |
| Hispanic/Latino(a) | 7,522 | 11.8% | 7,661 | 12.0% |
| International | 18,284 | 28.6% | 19,602 | 30.7% |
| Pacific Islander | 229 | 0.4% | 251 | 0.4% |
| White | 19,266 | 30.1% | 17,636 | 27.6% |
| **Sub-total** | **64,007** |  | **63,798** |  |
| **Total** | **294,536** |  | **294,205** |  |

Table 2. International Student Nationality in University of California in 2022^1^

| **Nationality** | **Students (N)** | **Percent Among International Students** |
| --- | --- | --- |
| **China** | 20,970 | 54% |
| **India** | 4,543 | 12% |
| **South Korea** | 1,994 | 5% |
| **Taiwan** | 1,429 | 4% |
| **Canada** | 1,013 | 3% |
| **Japan** | 592 | 2% |

Note: Other 159 countries had 1% or less student population (e.g., Indonesia, Iran, Vietnam, Turkey, Mexico, Brazil, UK, Singapore, Bangladesh, Thailand, Malaysia, etc.)

1. University of California. Fall enrollment at a glance. Fall enrollment at a glance. Accessed July 24, 2024. https://www.universityofcalifornia.edu/about-us/information-center/fall-enrollment-glance [↑](#footnote-ref-1)
