# Supplementary material for: Double jeopardy study protocol: mixed-methods study to understand ANHPI college students at the intersection of sexual violence and anti-Asian racism after COVID-19
Source: BMC Public Health. 2025 Dec 22;25:4275. doi: 10.1186/s12889-025-25533-8 (PMC12723919; doi:10.1186/s12889-025-25533-8)
Supplement: Supplementary file 2 — Supplementary Material 2 [file 12889_2025_25533_MOESM2_ESM.docx]

## Appendix 4. Semi-Structured In-Depth Interview Guide

**Welcome and Introductions:**

Hello (*participant’s name*). My name is ____________. I am (introduce yourself) and I am going to conduct this interview today. We can’t do this in person due to the pandemic, so thanks for joining me virtually today. I really appreciate your time. Let me tell you a little about the research project and what you can expect in this interview.

This interview is designed to gather information about your experiences as an ANHPI *(explain if necessary*) student. Through the interview, I will be asking you about your observations and experiences with incidents of unwanted sexual contact and discriminations you may have experienced. The goal of the Double Jeopardy Study is to open the conversation about Asian students’ experiences of sexual violence and discrimination before and during the pandemic and use evidence-based practice to improve services to help students access supportive resources.

The highlights of this interview are:

- We will meet for about 60 minutes.
- Participation in this interview is completely voluntary. You may decide to answer as many questions as you would like in as much or little detail as you are comfortable sharing. You may also decide not to answer questions. At any time, you are free to say you want to end the interview.
- I will be taking notes during the interview.
- I will be recording the interview even while I take notes because we don’t want to miss anything. For transcription purposes, only voice will be recorded with your consent.
- All the information you provide will be kept confidential by the UCLA research team. Your name or other identifying information will not be shared with anyone and will not be used in any reports that we write following this interview. If you would like to use a pseudonym during this interview, you may. If you want to use an alternative name, please introduce yourself to me with that name, or tell me what name you would like to use now.

You will receive a $50 Amazon e-gift card once we completed this interview.

Do you have any questions for me at this point? *(answer questions if applicable)*

Can I have your verbal consent to participate in this interview? *(Prompt the participate to say yes if agree)*

Can I have your verbal consent to record? *(Prompt the participate to say yes if agree)*

I will start recording on Zoom now, you will see a notification message showing on your screen.

**Semi-Structured Interview Questions**

**Section 1: Background (5mins)**

Now, I would love to hear a bit about you and your background.

*Prob: Can you describe your personal journey to this point (highlights- challenges, successes, milestones…)*

*Prob: What’s your family like?*

*Prob: How would you describe your life as a student?*

*(only for international students) Prob: How long have you been in the United States?*

*(only for international students) Prob: How’s your experience in the States so far?*

**Section 2: Discrimination & Sexual Violence Incidents (20-30mins)**

Now I want to begin talking about your experience with potential **discrimination** as an Asian student.

What’s your perception of discrimination in the United States?

1. Can you give me an example of a time that you felt uncomfortable due to potential discrimination against your identify?
   1. How did that make you feel?
2. How has it impacted your life?
   1. How has it impacted your academic life?
   2. How has it impacted your personal life?
   3. How has it impacted your mental or emotional health?

Now I’d like to talk about your experience with **sexual violence.** I just want to remind you that please let me know if you don’t feel comfortable sharing any of the details.

1. Can you give me an example of a time that you felt uncomfortable due to unwanted sexual contact?
   1. How often does this happen?
2. How does that make you feel?
3. How has it impacted your life?
   1. How has it impacted your academic life?
   2. How has it impacted your personal life?
   3. How has it impacted your mental or emotional health?

As you know, the **Covid-19 pandemic** has changed many things in this world….

1. How has Covid-19 affected your life as an Asian student?
2. How do you think the pandemic has changed your experience of discrimination?
3. How do you think the pandemic has changed the experience of sexual violence?

**Section 3: Help-Seeking Behaviors and Coping Mechanisms (10-15mins)**

**I want to thank you for sharing your experience with us. Now I would like to understand more about how you coped with the incident(s).**

1. How did you handle the incident?

2. What kind of support did you have?

3. What’s your understanding of on-campus services such as Title IX & CARE?

1. How would you think the involvement of these services may change the situation?
2. What kind of support did you wish you had back then?

**Section 4: Closing questions (less 5 mins)**

1. Now looking back to yourself as a survivor, can you describe something that you wish to change?
2. Is there something you may not have thought about before that occurred to you during our conversation?
3. Are there any more questions for me about this interview?

**Section 5: Soliciting interest in the Trnasmedia-Photovoice Study**

Thank you so much for sharing your experiences and insights with us today. As we conclude this interview, we would like to also invite you to participate in a transmedia project that aims to raise awareness about the experiences of student survivors of sexual violence. I am going to give you a little bit overview of the project and you can let me know if you are interested.

*(Proceed to the next, if the participant agrees)*

The goal of this transmedia project is to give voice to your narrative and create a platform to share your story with the public. We believe that by presenting your experiences through mediums such as photos, audio files, painting, poems, we can effectively convey the depth and impact of your experience as an ANHPI woman. We want to shed light on the unique challenges faced by student survivors, raising awareness and fostering understanding within the wider community.

*(You can show them the study website as an example)*

As a token of our appreciation for your time and contribution, we will compensate you for your involvement in the project. The compensation will be up to $150, depending on the level of participation and the format of your submission.

You can submit any form of media that you believe captures your identity and relates to your experience of sexual violence. This could include cultural items that hold personal significance, relevant photographs, audio or written narratives reflecting your emotions tied to a picture, meaningful poems, and more. We encourage you to be creative and express yourself in a way that resonates with you. If you are interested, one of our team members will contact you within a week via email. We will assist you in brainstorming ideas and provide support throughout your creation process.
