# Supplementary material for: Double jeopardy study protocol: mixed-methods study to understand ANHPI college students at the intersection of sexual violence and anti-Asian racism after COVID-19
Source: BMC Public Health. 2025 Dec 22;25:4275. doi: 10.1186/s12889-025-25533-8 (PMC12723919; doi:10.1186/s12889-025-25533-8)
Supplement: Supplementary file 3 — Supplementary Material 3 [file 12889_2025_25533_MOESM3_ESM.docx]

## Appendix 3. Interview Process and Pre/Post Memo Templates

A. Flowchart of the Double Jeopardy Study

B. Pre-Interview Memo

- Pseudonym of the participant:
- Interviewer:
- Interview Date:
- Use your five senses: how do you feel right now? What’s the environment surrounding you like right now? What can you see? What can you hear?
- What do you know about this participant?
  - What’s your assumption about the participant after reading the survey responses?

C. Post Interview Memo

- Pseudonym of the participant:
- Interviewer:
- Interview Date:

1. How did you feel about this interview and the participant in general?
2. What stood out to you in this interview? Share one or two things.
3. What patterns have you observed across interviews?
4. What, if any, contrasting experiences, ideas, etc. did you notice in this interview compared to others?
5. What, if any, topics, questions or prompts were not clearly understood by this interviewee? What, if any, language seemed to help clarify this for the interviewee? Please note any other difficulties you had with the interview guide.
