# Supplementary material for: Double jeopardy study protocol: mixed-methods study to understand ANHPI college students at the intersection of sexual violence and anti-Asian racism after COVID-19
Source: BMC Public Health. 2025 Dec 22;25:4275. doi: 10.1186/s12889-025-25533-8 (PMC12723919; doi:10.1186/s12889-025-25533-8)
Supplement: Supplementary file 4 — Supplementary Material 4 [file 12889_2025_25533_MOESM4_ESM.docx]

## Appendix 2. Trauma-Informed Research

The proposed research study focuses on understanding and preventing sexual assault, sexual harassment, dating violence, and other forms of sexual misconduct and abuse that occur in relationships and between intimate partners. As outlined in our application, we are *not* specifically recruiting participants based on their self-identification as a survivor, perpetrator or witness of abuse or violence. However, statistically speaking, it is likely that a proportion of our participants will have experienced some form of abuse/violence at one or more points in their lives, possibly even including during their time at UCLA. Although we will not be prompting anyone to speak about their own experiences of violence or abuse, it is possible that a participant could be distressed or upset by or could experience ‘trauma’ by thinking about these topics. In this document we address the following:

1. Trauma, defined
2. What does it mean to be trauma-informed?
3. Elements and principles of a trauma-informed approach
4. What is trauma-informed research?
5. How to conduct trauma-informed research
6. Trauma-informed research training
7. References

**1. TRAUMA, DEFINED:** According to the Trauma-Informed Care Implementation Resource Center (<https://www.traumainformedcare.chcs.org/>), trauma can result from “exposure to an incident or series of events that are emotionally disturbing or life-threatening with lasting adverse effects on the individual’s functioning and mental, physical, social, emotional, and/or spiritual well-being.” Experiences that may be traumatic include (but are not limited to): Physical, sexual, and emotional abuse; Childhood neglect or abuse; and Violence in the community.^1^

**2. WHAT DOES IT MEAN TO BE TRAUMA-INFORMED?** Being ‘trauma-informed’ refers to an approach that is used (either in research or the provision of medical care or behavioral health services, etc.) whereby the researcher or practitioner has an understanding of what trauma is (i.e., an experience or collection of experiences that cause(s) intense physical and psychological stress reactions, as noted above). Further, the researcher/practitioner also has a strong awareness of the impact trauma can have across settings, services, and populations. A trauma-informed approach is used with the goal of seeing the research participant / patient / client) first – as a whole person – as opposed to seeing them as person with a problem. Being trauma-informed is often described as the implementation of techniques to shift focus away from asking research participants or medical patients or counseling clients *“What’s wrong with you?”* (which implies that the person is somehow ‘broken’ or ‘damaged’ or ‘problematic’) to *“What happened to you?”* (which recognizes that both social determinants and context play significant roles in how individuals perceive and process traumatic events, whether they are acute or chronic). Being trauma-informed involves viewing (trauma) through an ecological and cultural lens and recognizing that many factors (at many different levels and across the lifespan) can contribute to how someone who has had experiences that cause intense physical and psychological stress identifies and responds to their trauma.^1-3^

**3. ELEMENTS AND PRINCIPLES OF A TRAUMA-INFORMED APPROACH:** The Substance Abuse and Mental Health Services Administration (SAMHSA: <https://www.samhsa.gov/>) identified three key elements and six principles of a trauma-informed approach that are shown in Box 1.

| **BOX 1: SAMHSA’s Key Elements and Principles of a Trauma-Informed Approach^2,3^** | |
| --- | --- |
| **Elements of being trauma-informed** | **Principles of being trauma-informed** |
| Referred to as the ***THREE “E’S” OF TRAUMA***: Event(s), Experience of Event(s) and Effect are considered the key elements of a trauma-informed approach, specifically:   1. **Event(s):** This refers to understanding what the event(s) is/are (i.e., their definition) and what their prevalence is. 2. **Experience of Event(s):** This refers to understanding an individual’s experience and determining if trauma has occurred. 3. **Effect** is recognizing how trauma affects all individuals involved; and responding by knowledge of all E’s into practice. | These principles are not meant to serve as a prescribed set of practices or procedures but as factors that may be generalizable across different settings and situations. They are:   1. Safety 2. Trustworthiness and Transparency 3. Peer Support 4. Collaboration and Mutuality 5. Empowerment, Voice and Choice 6. Cultural, Historical, and Gender Issues |

**4. WHAT IS TRAUMA-INFORMED RESEARCH?** The goal of trauma-informed research is to avoid re-traumatizing someone by inadvertently recreating or bringing up memories of conditions of a persons’ previous trauma(s) which, in turn, can cause the reliving of trauma in the moment (for example, during a focus group discussion where availability of sexual violence services is brought up).^1-3^ A trauma-informed approach to conducting research requires that all members of the research team are knowledgeable about the topic being investigated. The researchers should understand the prevalence of the different forms of abuse and violence being studied, overall and in the specific population being examined. Research team members should also have a firm understanding of the determinants of these different types of traumatic events, and what their consequences are. Further, the researchers must know how to provide short-term mechanisms of support to research participants who may be in need, and they must also have comprehensive information available to provide to participants on where they can go to receive more in-depth services. Adopting trauma-informed practices such as these aims to improve participant engagement in services, if and when needed, which will, hopefully, improve health outcomes and contribute to future prevention.^2-3^

**5. HOW TO CONDUCT TRAUMA-INFORMED RESEARCH:** Trauma-informed research activities include mechanisms that enable researchers to establish feelings of safety and trust with the research participants. If these feelings can be established, participants will be more open to sharing information and perspectives and to collaboratively engage in conversation. How did it come about? The Trauma-Informed Care Implementation Resource Center (<https://www.traumainformedcare.chcs.org/>) developed specific interviewing techniques for conducting trauma-informed research interviews. These are shown in Box 2.

**BOX 2: Trauma-Informed Approaches to Interviewing^*^**


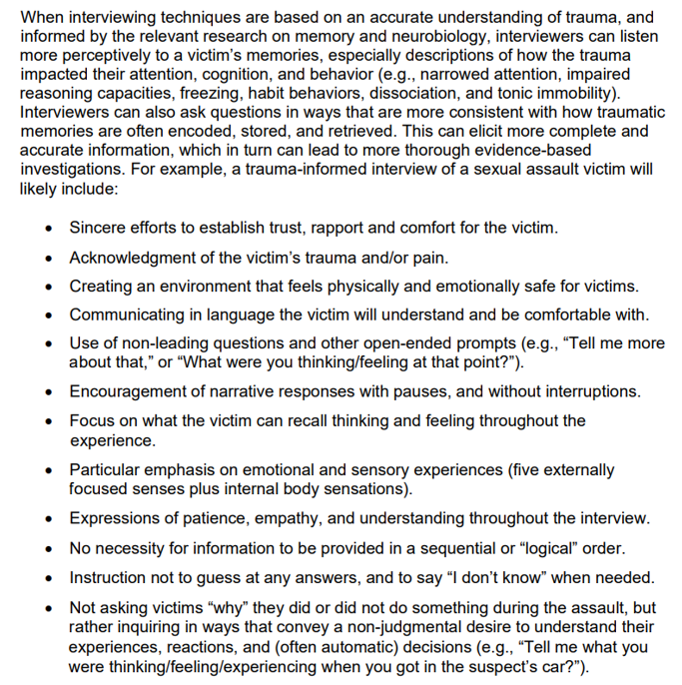


*Copied directly from pages 7-8 of: Lonsway, K.A., Hopper, J., Archambault, J. (2020). Becoming Trauma Informed: Understanding and Appropriately Applying the Neurobiology of Trauma. End Violence Against Women International.

**6. TRAUMA-INFORMED RESEARCH TRAINING:** In addition to completing the standard research, ethics, and compliance training through the UCLA Collaborative Institutional Training Initiative (CITI Program), all research team members will receive new or refresher training on trauma-informed research and care, as well as the safe and ethical conduct of research on violence against women. Specific considerations and procedures to be followed are detailed below.

1. **All members of the research team must be carefully selected and trained** per recommendations developed for the World Health Organization (WHO) from those prepared for the WHO Multi-Country Study on Women’s Health and Domestic Violence.^4^
2. **Specialized training for all research team members.** Also drawing from the WHO’s Ethical and Safety Recommendations for Research on Violence,^4^ all team members will receive new or refresher training and on-going support *“over and above that normally provided to research staff.”* ^4, page 19^ Such training will include:
   1. *Training on different forms of violence; orientation to the concepts of gender, gender discrimination and gender inequality.* Training and ongoing discussion will address how to confront and overcome any of our own biases, fears and stereotypes regarding survivors, perpetrators and/or witnesses of violence. This training will be facilitated by the PI.
   2. *Training on the neurobiology of trauma, trauma-informed care and UCLA and community resources for survivors.* This training will be conducted in multiple workshops provided by professionals from UCLA Campus Assault Resources & Education (CARE), <https://careprogram.ucla.edu/>) which provides comprehensive outreach and prevention education to the campus community on sexual assault, dating, and domestic violence, and stalking. All members of our team will participate in at least two “custom workshops” led by the CARE Prevention Education Coordinator, Elizabeth Rogers ([erogers@careprogram.ucla.edu](mailto:erogers@careprogram.ucla.edu)) who will lead us through discussion on the root causes of violence, focusing specifically on:
      1. An overview of CARE services,
      2. The effects of trauma,
      3. How to support survivors and provide trauma-informed care,
      4. The neurobiology of trauma.
   3. *Workshop(s) and other activities for research team members to come to terms with their own experiences with abuse or deal with the consequences of hearing about abuse of others.* Several members of our team have had their own experiences with violence, abuse and/or discrimination. We recognize that this increases our individual and collective passion for addressing these topics and finding solutions for prevention. We also recognize these experiences and passions can increase and bolster our skills as researchers, as well as our levels of empathy and understanding toward one another, as well as our participants. At the same time, the process of being involved in a study (either as an interviewer, coordinator, supervisor, data manager, etc.) focused on violence can create emotional challenges, including being triggered or feeling depressed or re-traumatized (either directly or vicariously). As such, we will have the following activities to address the emotional needs of the team.
      1. A workshop with a psychosocial counselor, who focuses on trauma and family and interpersonal violence, to discuss vicarious trauma and self-care.
      2. Ongoing debriefing meetings to enable all members of the research team to discuss what they are hearing, their feelings about the situation, and how it is affecting them. The goal of these meetings is to reduce the stress of the fieldwork, and avert any negative consequences.
      3. All team members will have the open and ongoing opportunity to discuss any of their experiences in private with the PI.
      4. Access to UCLA Counseling and Psychological Services.
3. **Other ethical and safety recommendations of relevance.** Of the remaining WHO Ethical and Safety Recommendations for Research on Violence,^4^ five are of much relevance to the proposed study and are outlined in Box 3, below. All will be adhered to in the proposed research.

**BOX 3: The Other WHO Ethical and Safety Recommendations for Violence Research that are Most Relevant to the Proposed Study**

1. The safety of research participants and the research team is paramount, and should guide all project decisions.
2. Protecting confidentiality is essential to ensure participants’ safety and data quality.
3. The study design must include actions aimed at reducing any possible distress caused to the participants by the research.
4. Fieldworkers should be trained to refer any participant requesting assistance to available local services and sources of support.
5. Researchers and donors have an ethical obligation to help ensure that their findings are properly interpreted and used to advance policy and intervention development.

**7. REFERENCES**

1. Lonsway, K.A., Hopper, J., Archambault, J. (2020). Becoming Trauma Informed: Understanding and Appropriately Applying the Neurobiology of Trauma. End Violence Against Women International.
2. Substance Abuse and Mental Health Services Administration. Trauma-Informed Care in Behavioral Health Services. Treatment Improvement Protocol (TIP) Series 57. HHS Publication No. (SMA) 13-4801. Rockville, MD: Substance Abuse and Mental Health Services Administration, 2014.
3. Substance Abuse and Mental Health Services Administration. SAMHSA’s Concept of Trauma and Guidance for a Trauma-Informed Approach. HHS Publication No. (SMA) 14-4884. Rockville, MD: Substance Abuse and Mental Health Services Administration, 2014.

Putting women first: ethical and safety recommendations for research on domestic violence against women. Geneva: World Health Organization; Available from: https://apps.who.int/iris/handle/10665/65893
