# Supplementary material for: Double jeopardy study protocol: mixed-methods study to understand ANHPI college students at the intersection of sexual violence and anti-Asian racism after COVID-19
Source: BMC Public Health. 2025 Dec 22;25:4275. doi: 10.1186/s12889-025-25533-8 (PMC12723919; doi:10.1186/s12889-025-25533-8)

## Appendix 6. Art Exhibit and Panel Discussion to Engage with Student Community

Our team curated a compelling exhibition and panel discussion at the UCLA campus, with more than 120 attendees. Featuring diverse artworks and interactive exhibits, we ignited vital conversations and heightened awareness about on-campus sexual violence. This event showcased passionate student activists and experts from UCLA, as well as student organizations like Activism Through Policy and Bruin Consent Coalition. Furthermore, our outreach within our home institution expanded through fruitful collaborations with other campuses in Southern California, including University of Southern California (USC), Occidental College, Pomona College, and Claremont McKenna College. It has fostered a holistic and inclusive approach to our project's objectives, leaving a lasting legacy of unity, art, and activism in the pursuit of a safer and more inclusive college environment.

Date: April 23, 2024

Location: Los Angeles, California, USA

Picture 1. Fabric art of quotes


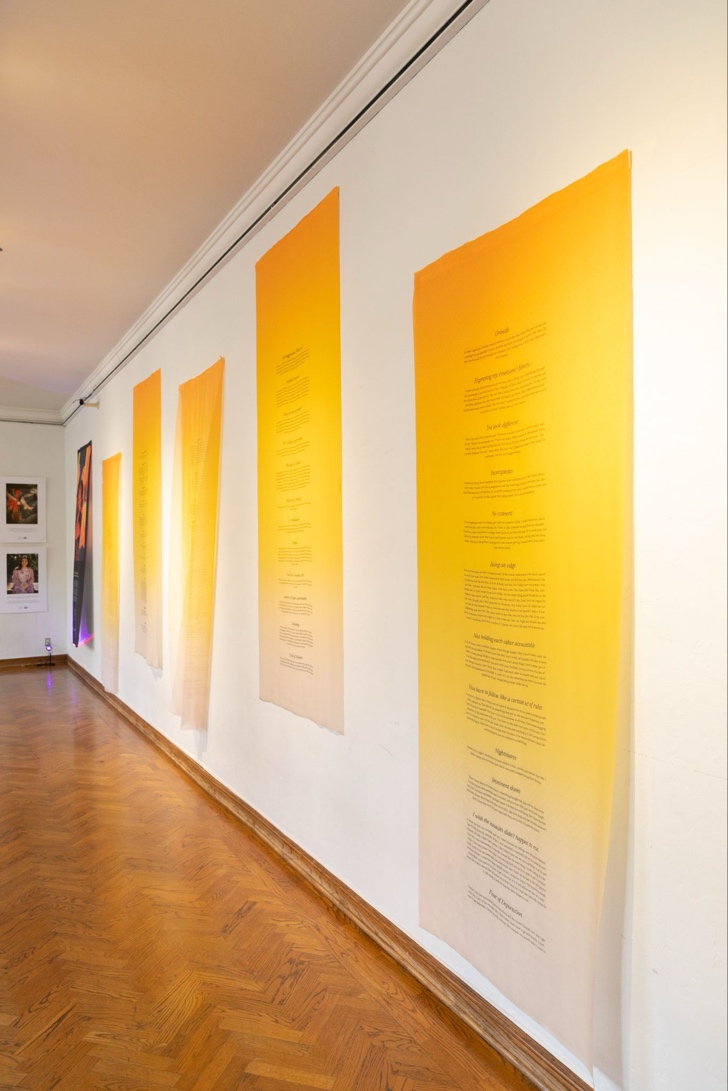


Picture 2. Fabric art of quotes


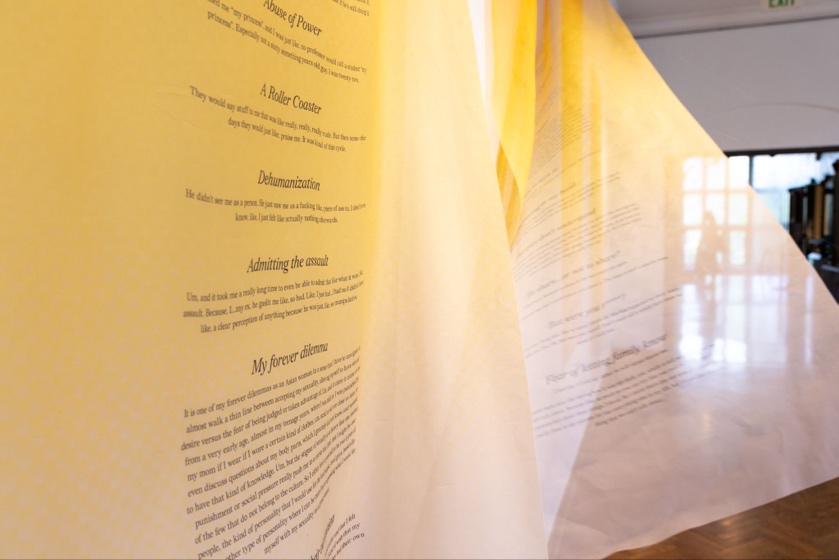


Picture 3. Community exhibition at UCLA


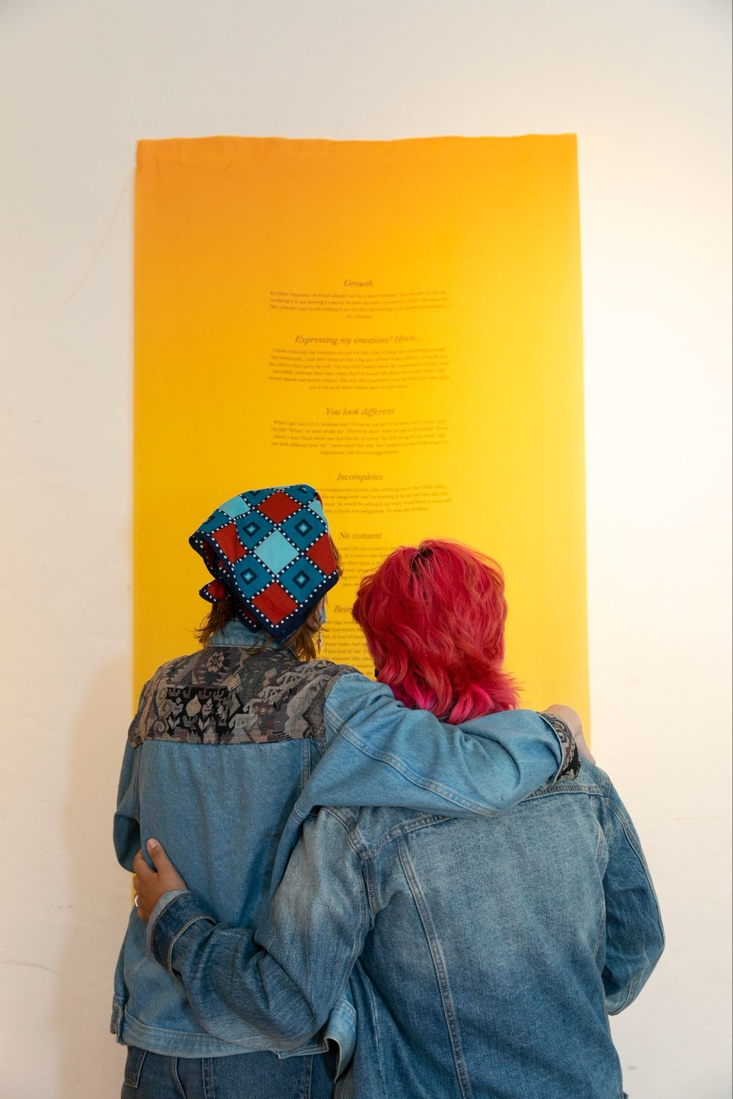


Picture 4. Community exhibition at UCLA


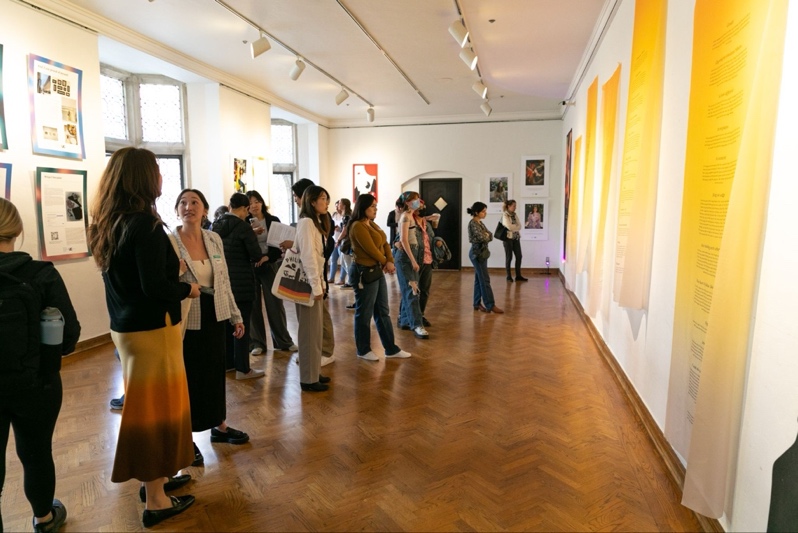


Picture 5. Community discussion at UCLA


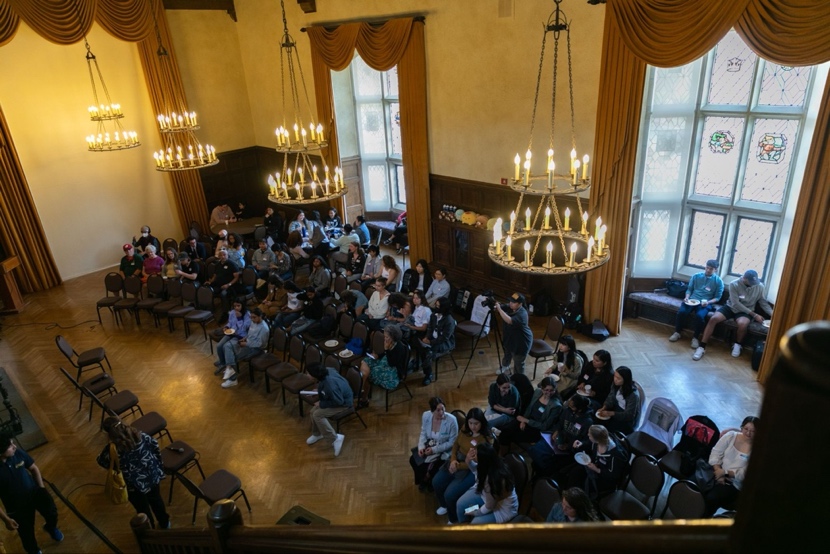


Picture 6. Panel discussion at UCLA


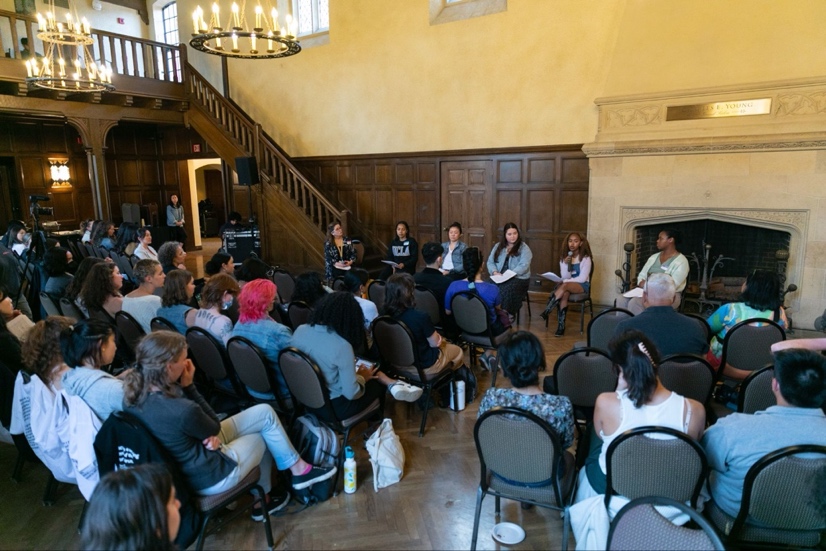

Supplement: Supplementary file 5 — Supplementary Material 5 [file 12889_2025_25533_MOESM5_ESM.docx]
