# Supplementary material for: Double jeopardy study protocol: mixed-methods study to understand ANHPI college students at the intersection of sexual violence and anti-Asian racism after COVID-19
Source: BMC Public Health. 2025 Dec 22;25:4275. doi: 10.1186/s12889-025-25533-8 (PMC12723919; doi:10.1186/s12889-025-25533-8)
Supplement: Supplementary file 6 — Supplementary Material 6 [file 12889_2025_25533_MOESM6_ESM.docx]

## Appendix 5. Transmedia Artwork

Picture 1. Illustration and essay titled "Something I can hold"


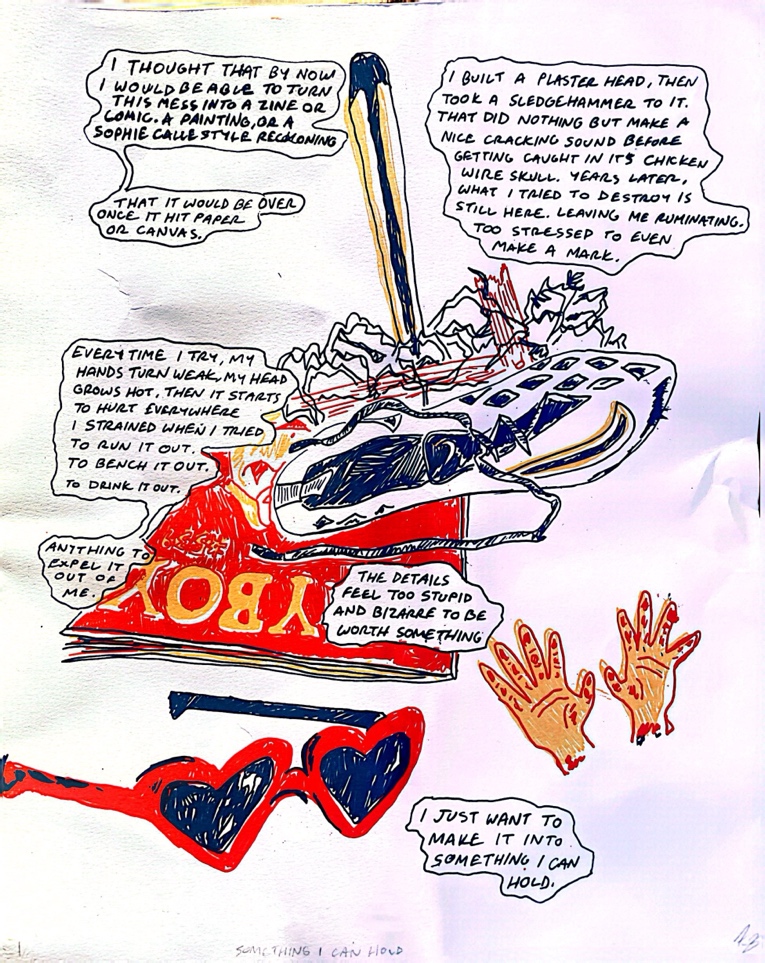


I thought that by now I would be able to turn this mess into a zine or comic, a painting, or a Sophie Calle style reckoning.

That it would be over once it hit paper or canvas.

I built a plaster head, then took a sledgehammer to it. That did nothing but make a nice cracking sound before getting caught in its chicken wire skull. Years later, what I tried to destroy is still here. Leaving me ruminating, too stressed to even make a mark.

Every time I try, my hands turn weak, my head grows hot, then it starts to hurt everywhere I strained when I tried to run it out. To bench it out. To drink it out.

Anything to expel it out of me.

The details feel too stupid and bizarre to be worth something.

I just want to make it into something I can hold.

Picture 2. Photography and essay


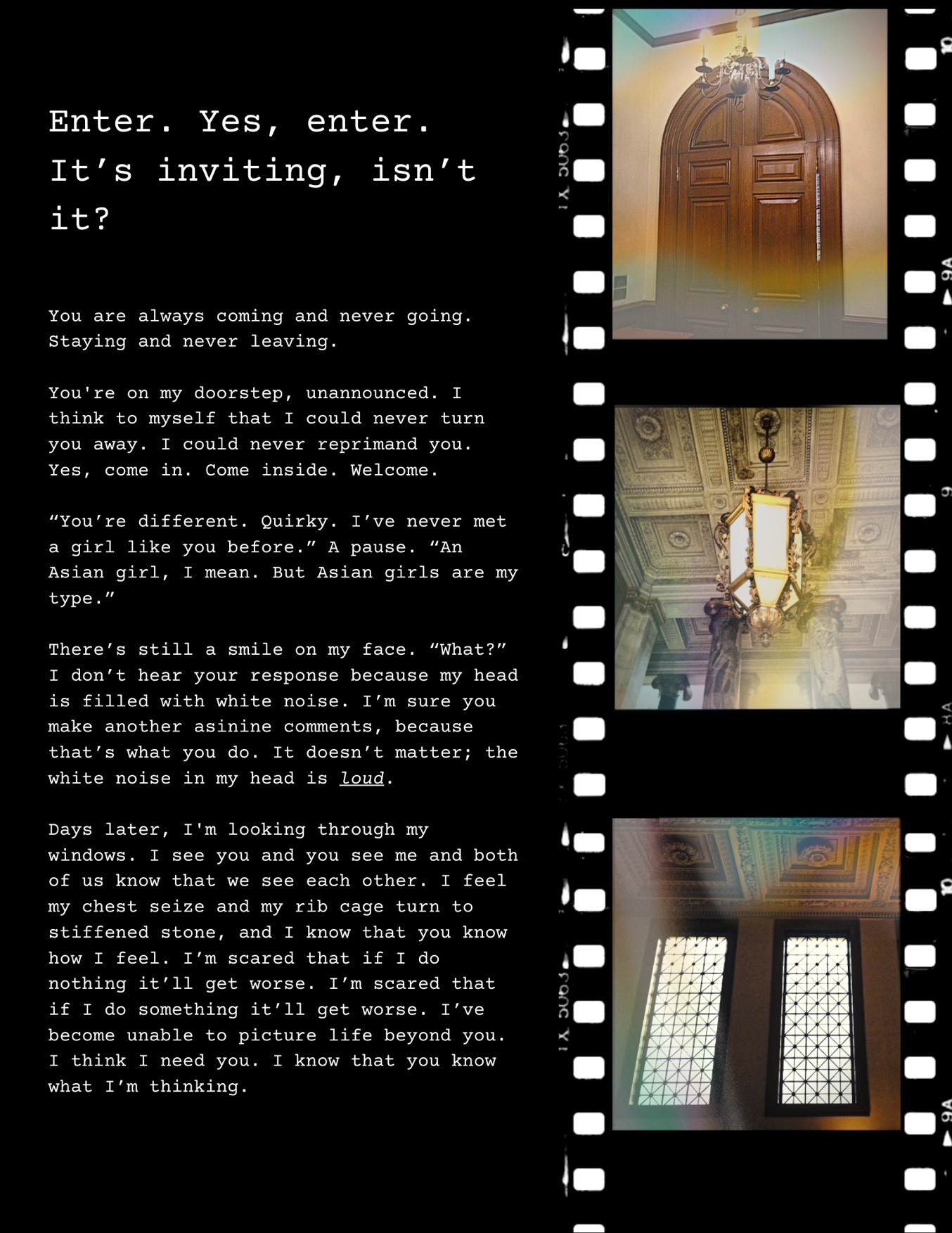


Picture 3. Photography and essay

**
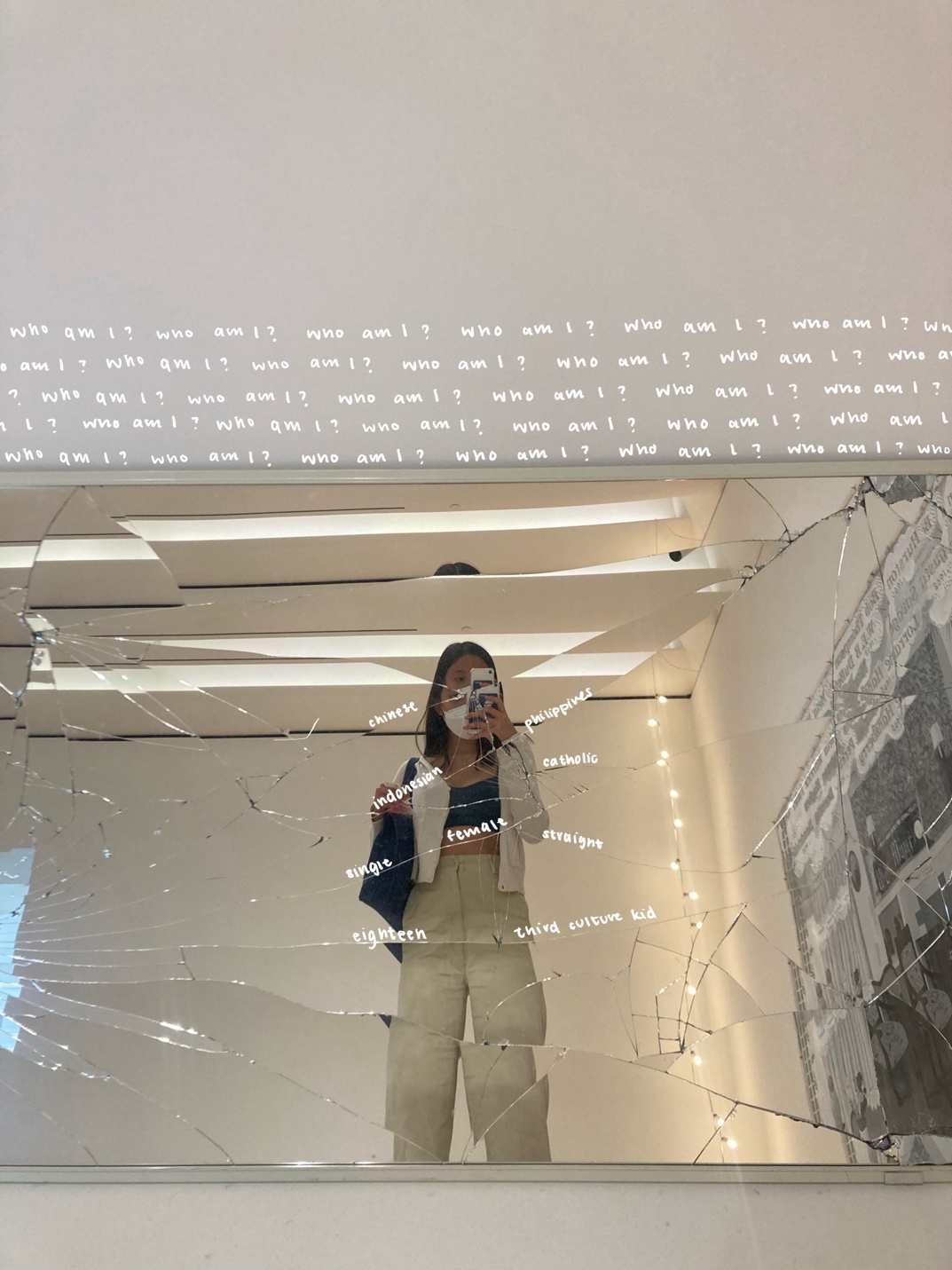
**

*“I am no longer shoving my traumatic experiences into the dark corners of my brain. Instead, I place my experiences on a pedestal. A bright, beaming light ironically illuminating and spotlighting all my dark, negative memories.”*

*“I carefully examine my trauma, inspecting the damages from the accumulation of these experiences. I observe the ripples it has caused in my life and how it has shaped who I am today: my fears, aspirations, desires, and dreams. After keenly assessing my trauma, I excitedly flash and wave a middle finger in the face of my past experiences. I decide to move on. I choose to forgive but to never, ever forget. I don’t forget the drops of blood on scissors as I score my skin to exchange my emotional pain for a physical one. I don’t forget the suffocating feeling of alienation, choking me so tightly that I failed to ask for help as I plunged farther into an abyss of darkness. I don’t forget the immense feeling of shame and regret surrounding me after being toyed with and treated as a sex toy. I don’t forget that a banana is 100 calories, a chicken nugget is 60 calories, and an egg is 80 calories. I don’t forget the sleepless nights when my brain constantly replays countless ruminating thoughts about how much I hated myself. I don’t forget using alcohol and weed as a coping mechanism as I desperately run away from the problems that haunt me.”*

*“Remembering these experiences has allowed me to learn, grow, and flourish. I have learned how to embrace authenticity: to remain myself regardless of the situations thrown at me. I have learned to stop dismissing negative emotions with false reassurances; Instead, I respond to and validate these emotions. I have become stronger and more resilient. I am not letting myself be a puppet controlled by the experiences I had in the past. I am not locking myself in a vicious cycle of negative thoughts. I want to live in the present, not ruminate in the past. I want to love, not hate. And, I am proud of myself.”*
